# Multifunctional Au@AgBiS_2_ Nanoparticles as High‐Efficiency Radiosensitizers to Induce Pyroptosis for Cancer Radioimmunotherapy

**DOI:** 10.1002/advs.202302141

**Published:** 2023-09-08

**Authors:** Liang Xiao, Benjin Chen, Wanni Wang, Tian Tian, Haisheng Qian, Xiaohu Li, Yongqiang Yu

**Affiliations:** ^1^ Department of Radiology Research Center of Clinical Medical Imaging Anhui Province Clinical Image Quality Control Center The First Affiliated Hospital of Anhui Medical University Hefei Anhui 230022 P. R. China; ^2^ Department of Pharmacology School of Basic Medical Sciences Anhui Medical University Hefei 230032 P. R. China; ^3^ School of Biomedical Engineering Anhui Provincial Institute of Translational Medicine Anhui Engineering Research Center for Medical Micro‐Nano Devices Anhui Medical University Hefei 230011 P. R. China; ^4^ Department of Oncology The First Affiliated Hospital of Anhui Medical University Hefei Anhui 230036 P. R. China

**Keywords:** core‐shell structures, DNA damage and repair, pyroptosis, radioimmunotherapy, radiosensitization

## Abstract

Radiotherapy (RT), a widely used clinical treatment modality for cancer, uses high‐energy irradiation for reactive oxygen species (ROS) production and DNA damage. However, its therapeutic effect is primarily limited owing to insufficient DNA damage to tumors and harmful effects on normal tissues. Herein, a core‐shell structure of metal–semiconductors (Au@AgBiS_2_ nanoparticles) that can function as pyroptosis inducers to both kill cancer cells directly and trigger a robust anti‐tumor immune against 4T1 triple‐negative murine breast cancer and metastasis is rationally designed. Metal‐semiconductor composites can enhance the generation of considerable ROS and simultaneously DNA damage for RT sensitization. Moreover, Au@AgBiS_2_, a pyroptosis inducer, induces caspase‐3 protein activation, gasdermin E cleavage, and the release of damage‐associated molecular patterns. In vivo studies in BALB/c mice reveal that Au@AgBiS_2_ nanoparticles combined with RT exhibit remarkable antitumor immune activity, preventing tumor growth, and lung metastasis. Therefore, this core‐shell structure is an alternative for designing highly effective radiosensitizers for radioimmunotherapy.

## Graphical abstract

Au@AgBiS_2_ core‐shell nanoparticles have been successfully fabricated using an intermediate layer conversion method to enhance DNA damage and facilitate substantial production of reactive oxygen species (ROS), resulting in enhance anti‐tumor therapeutic efficacy via pyroptosis to kill cancer cells both directly and indirectly through immunoactivation under irradiation conditions.

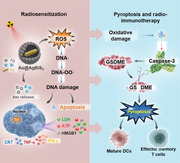

## 1 Introduction

Radiotherapy (RT) is an important treatment modality for malignant tumors. Approximately 70% of patients with cancer have received RT during the treatment course.^[^ ^1^ ^]^ Radiotherapy is primarily characterized by direct DNA damage and indirect reactive oxygen species (ROS) production via water radiolysis, resulting in ionizing radiation (X‐rays/γ‐rays)‐induced cancer cell apoptosis.^[^ ^2^ ^]^ However, it is extremely challenging to completely eliminate tumors owing to the complex tumor microenvironment (TME) and unpredictable response of surrounding normal tissues.^[^ ^3^ ^]^ Furthermore, the maximum RT dose that tumors can receive is limited by normal tissue tolerance. In addition, higher radiation doses are frequently impractical, resulting in the emergence of more adverse effects in healthy tissues.^[^ ^4^ ^]^ Therefore, the dose used in clinical RT is often a tradeoff between tumor efficacy and radiation damage. The strategies to enhance radiosensitivity are mainly based on the physical and chemical features and sensitization mechanisms of radiation to obtain safe and efficient radiosensitizers under low‐dose conditions.^[^ ^5^ ^]^ Recently, nanomaterials based on high atomic number (Z) elements such as Au, Ag, Bi, and Gd and some semiconductors have emerged as potential radiosensitizers by effectively depositing more radiation energy into tumor areas.^[^ ^6^ ^]^ However, ideal radiosensitizers exhibit excellent biosafety and selective tumor accumulation, decreasing the probability of complications in normal tissues. According to our previous study, hollow AgBiS_2_ nanospheres can induce tumor cell‐specific toxicity but not induce any toxicity to normal cells, suggesting their tumor‐targeting ability and biosafety.^[^ ^7^ ^]^

Notably, RT not only causes DNA and oxidative damage directly and indirectly but also induces tumor antigen production to enhance antitumor immune responses; this is vital for radiation‐induced tumor remission.^[^ ^8^ ^]^ The robust immune response between tumors and stromal cells in the TME is associated with X‐ray irradiation, which can specifically lead to cancer cell death via the synergistic effects of RT and immunotherapy.^[^ ^9^ ^]^ However, when using this combination strategy, maintaining a boosted immune response and long‐term immune memory is challenging.^[^ ^10^ ^]^ Therefore, developing new methods to increase RT efficacy and produce a sustainable immune memory response is urgently warranted. Pyroptosis is characterized by the generation of membrane pores and swelling of cells with large bubbles. Immune responses are triggered by the leakage of inflammasome molecules and cell contents into the extracellular fluid. During this process, a superfamily of proteins called gasdermin, including gasdermin A–E, exhibits perforation capabilities and plays a vital role.^[^ ^11^ ^]^ In 2017, Shao et al. reported that gasdermin E (GSDME) is cleaved and activated by caspase3 and explored a new mechanism underlying chemotherapeutic drug‐induced pyroptosis for the first time.^[^ ^12^ ^]^ In 2020, Liu and Sun et al. revealed that pyroptosis can not only effectively kill tumor cells but also induce antitumor immune activity by triggering strong inflammatory responses.^[^ ^13^ ^]^ Moreover, Lin et al. reported that biodegradable nanoparticles (NPs) as pyroptotic inducers in tumor immunotherapy augment ROS production and caspase‐1 and 3 protein activation, further cleaving gasdermin D (GSDMD) and GSDME by disrupting intracellular and extracellular homeostasis.^[^ ^14^ ^]^ Specific ions, molecules, chemotherapeutic agents, such as ion, metformin, cisplatin, doxorubicin as well as high‐dose RT (10 Gy, single fraction) can induce GSDMD‐ or GSDME‐mediated pyroptosis.^[^ ^15^ ^]^ Taken together, these findings suggest that pyroptosis, triggered by excessive ROS production, in turn triggers a robust antitumor immune response and effectively inhibits lung metastasis by promoting long‐term immune memory.^[^ ^16^ ^]^ Therefore, it is a novel concept and broadens the scope of cancer treatment strategies.

**Figure 1:**
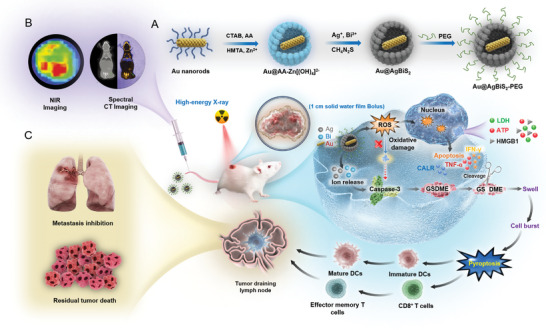
Schematic illustration of Au@AgBiS_2_^‐PEG^ design and anti‐tumor application. Descriptions provided for the indicated panels (A–C). The synthesis of multifunctional Au@AgBiS_2_ core‐shell structures using the intermediate layer conversion method (A). This composite exhibits the high efficacy as a radiosensitizer owing to the high level of ROS production and as an excellent pyroptosis inducer for boosting antitumor immunity (B) and effectively preventing lung metastasis (C).

In the present study, we fabricated a metal‐semiconductor core‐shell nanostructure of Au@AgBiS_2_ using the hard template‐engaged polyol method^[^ ^17^ ^]^ as an excellent radiosensitizer with good biosafety for improving RT efficacy in the TME and inducing pyroptosis to enhance both local and systemic anti‐tumor effects (**Figure** **1**). Under the irradiation of high‐energy X‐rays, the Bi elements as shell, as a high atomic number deposited more radiation energy, and Ag ions were released in small amounts, which will stimulate the production of immune response. On the other hand, Au nanorod as inner core remained a stable structure and continued to sensitize RT in the tumor microenvironment. The presence of high atomic number after RT treatment causes a large amount of ROS production and simultaneously induces pyroptosis to tumor cells, triggering a strong immune response to treat the primary tumor as well as effectively preventing lung metastasis.

## 2 Results and Discussion

### 2.1 Synthesis, Modification, and Characterization of Au@AgBiS_2_

**Figure 2:**
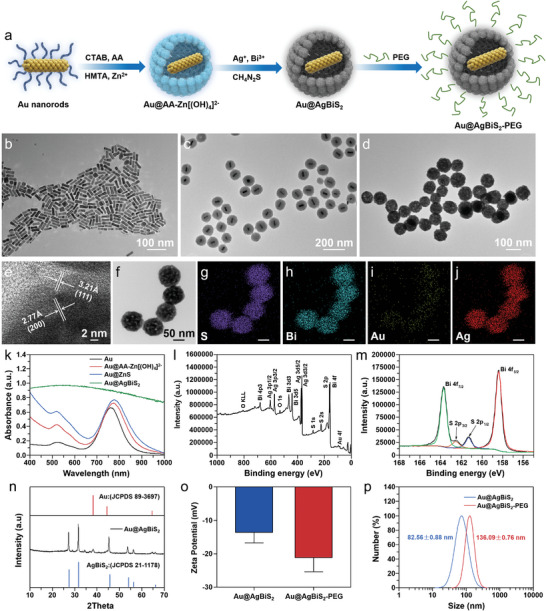
Synthesis, modification, and characterization of Au@AgBiS_2_. a) The synthesis process of the Au@AgBiS_2_. b–d) TEM images of the Au NRs, Au@ZnO, and Au@AgBiS_2_. e) HRTEM image of Au@AgBiS_2_. f–j) STEM images of Au@AgBiS_2_ and the elemental mappings of S, Bi, Au, and Ag, scale bars for g–j, 50 nm. k) UV–vis‐NIR absorbance spectra of Au, Au@AA‐[Zn(OH)_4_]^2−^, Au@ZnS, and Au@AgBiS_2_. l) XPS of the element peaks of Au@AgBiS_2_ and m) Bi 4f and S 2p peaks. n) XRD patterns of Au@AgBiS_2_. o) Zeta potentials and p) size distributions (n = 3) of Au@AgBiS_2_ and Au@AgBiS_2_‐PEG as determined by Malvern Zetasizer Nano ZS90.

In the present study, Au@AgBiS_2_ core‐shell NPs were prepared using the modified intermediate layer conversion method.^[^ ^17^ ^]^ As demonstrated in **Figure** **2**a, Au nanorods (Au NRs) were fabricated as described previously,^[^ ^18^ ^]^ with a uniform length of 43.8 ± 3.6 nm and diameter of 12.1 ± 1.9 nm (aspect ratio of ≈3.6:1; Figure 2b). As demonstrated in Figure 2c and Figure S1, S2 (Supporting Information), the core‐shell nanostructures were clear (average size of 94 ± 4.3 nm and shell thickness of 35.6 ± 2.1 nm), suggesting Au NR coating to form Au@AA‐[Zn(OH)_4_]^2−^ and Au@ZnS core‐shell nanostructures as previously described.^[^ ^19^ ^]^ Next, Au@AgBiS_2_ NPs were prepared using Au@ZnS NPs as template and adding Bi and Ag sources, which had uniform and discrete morphology as shown in a typical TEM (transmission electron microscopy) image (Figure 2d). The edge region of nanoparticles was selected to collect representative high‐resolution TEM image (HRTEM; Figure 2e). Well‐resolved lattice spacings of 0.321 and 0.277 nm corresponded to the (111) and (200) plane of AgBiS_2_, respectively. Figure 2f–j showed the Scanning transmission electron microscopy (STEM) image and elemental mappings of Au@AgBiS_2_ NPs, indicating there exist S, Bi, Au, and Ag elements. Besides, (UV–vis‐NIR) absorption spectra of Au, Au@ZnS, and Au@AgBiS_2_ was measured and the change in the spectral peak symbolized the change in the product obtained at each step of the synthesis process (Figure 2k). X‐ray photoelectron spectra (XPS) technology was also characterized to examine the composition of Au@AgBiS_2_ NPs (Figure 2l,m and Figure S3, Supporting Information). The band gap (E_g_) of AgBiS_2_ was obtained by UV–vis absorption spectra with the Tauc analyses, which was determined to be 1.1 eV (Figure S4a, supporting information). Ultraviolet photoelectron spectroscopy (UPS) was employed to investigate the band structure of Au@AgBiS_2_ (Figure S4b–d, supporting information). The electrons from AgBiS_2_ would transfer to Au at the interface until the E_F (AgBiS2)_ and E_F (Au)_ reached equilibrium, resulting in the new E_F_ generation. This process would leave a positive charge layer on the surface of AgBiS_2_ and lead to an accumulation of negative electrons in the surface of Au, giving rise to the deformation of band structure between the AgBiS_2_ and the Au, where the surface potential barrier (Schottky barrier) is formed.^[^ ^20^ ^]^ The peaks at 374.18, 368.08, 163.83, 158.48, 88.43, and 84.78 eV were associated with the binding energies of Ag 3d_5/2_, Ag 3d_3/2_, Bi 4f_7/2_, Bi 4f_5/2_, Au 4f_7/2_, and Au 4f_5/2_, respectively. The powder X‐ray diffraction (XRD) pattern of core‐shell nanostructures was investigated as shown in Figure 2n. The peaks located at 38.16°, 44.38°, and 64.66° can be indexed to the diffraction peaks of (111), (200), and (220) plane of cubic Au (JCPDS No. 89–3697). Additionally, the other sharp peaks were associated with the cubic phase of AgBiS_2_ (JCPDS No. 21–1178). Taken together, the abovementioned characterization results suggest the successful synthesis of Au@AgBiS_2_ NPs. To improve biocompatibility for further biomedical applications, as‐obtained Au@AgBiS_2_ NPs were modified with PEG‐C_18_PMH (noted as Au@AgBiS_2_‐PEG).^[^ ^21^ ^]^ The ζ‐potential value of Au@AgBiS_2_ NPs slightly changed from −13.57 ± 3.187 mV before modification to −21.16 ± 4.228 mV after modification (Figure 2o). Fourier transform infrared analysis (FTIR) was further used to confirm the successful preparation of Au@AgBiS_2_‐PEG. As shown in the following Figure, the peak located at ≈1650 and ≈1200 cm^−1^ were corresponding to the amide bond (C = O) and carbon‐oxygen single bond (C‐O‐C) of PEG‐C18PMH, suggesting the successful modification of Au@AgBiS_2_‐PEG (Figure S5, Supporting Information). Dynamic light scattering revealed that the average hydrodynamic diameter of Au@AgBiS_2_‐PEG was 136.09 ± 0.76 nm (Figure 2p); this is larger than that of pure Au@AgBiS_2_ (82.56 ± 0.88 nm), verifying the successful PEG modification.

### 2.2 Au@AgBiS_2_‐PEG can Potentiate DNA Damage and Prevent its Repair and Sensitized RT Via the Remarkable Production of ROS In Vitro

**Figure 3:**
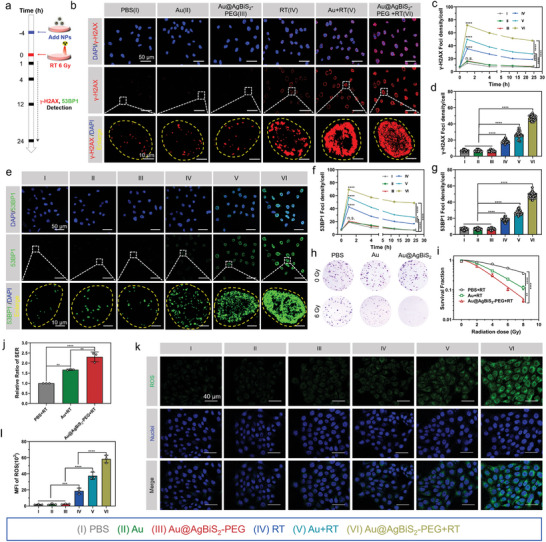
Au@AgBiS_2_‐PEG augmented RT‐mediate DNA damage and enhanced cancer radiosensitivity via ROS burst. a) Treatment planning for 4T1 cells pretreated with Au@AgBiS_2_‐PEG combined with RT on DNA damage and repair. 4T1 cells were incubated with PBS, Au or Au@AgBiS_2_‐PEG and administrated to a single dose of 6 Gy exposure for 1 h and observed with γ‐H_2_AX and 53BP1 antibodies by immunofluorescence staining after radiotherapy. b,e) γ‐H_2_AX foci (red) and 53BP1 foci (green) were detected by CLSM in cell nuclei (blue) of 4T1 cells. c,f) The result of γ‐H_2_AX foci and 53BP1 foci density (γ‐H_2_AX and 53BP1 foci per cell, n = 50 cells) of 4T1 cells after RT 6 Gy. d,g) The density of γ‐H_2_AX foci and 53BP1 foci in 4T1 cells at 24 h after 6 Gy exposure. h) Typical images of colony formation of 4T1 cells treated with PBS, Au or Au@AgBiS_2_‐PEG at various concentrations combined with RT 6 Gy. i) Survival fraction (SF) of 4T1 cells pretreated the different concentrations of PBS, Au or Au@AgBiS_2_‐PEG under various treatment conditions. j) The SER values of each treatment group were calculated by single‐hit multitarget model (n = 3). k) Generation of ROS within 4T1 cells with different pretreatment by CLSM observation. l) Calculation of mean fluorescence intensity (MFI) of ROS was detected by ImageJ based on DCFH‐DA (green) as probe and DAPI‐labeled cell nuclei (blue) in 4T1 cells (n = 3). Data are indicated as the mean ± SD, and analyzed with one‐way analysis of variance (ANOVA) (*P < 0.05, **P < 0.01, ***P < 0.001, ****P < 0.0001).

It has been reported that gold nanoparticles exhibit a significant dose‐dependent radiosensitization effect.^[^ ^22^ ^]^ Because gold nanoparticles are able to deposit more ionizing radiation energy during radiation therapy and trigger the generation of photoelectrons and Auger electrons, which can interact with water and oxygen to produce active free radicals that cause cell damage and radiosensitization effect. Moreover, Bi element with high atomic number also have excellent radiosensitization performance.^[^ ^23^ ^]^ We first investigated the radiosensitization effect of Au@AgBiS_2_‐PEG in vitro. Phosphorylated histones H_2_AX (γ‐H_2_AX) and P53‐binding protein 1 (53BP1) were used to characterize the kinetics of DNA damage repair.^[^ ^24^ ^]^ Murine triple negative breast cancer (4T1) cells were incubated with PBS, Au‐NRs, and Au@AgBiS_2_‐PEG with a single fraction of 6 Gy at different time intervals. Then the 4T1 cells were immunostained with γ‐H_2_AX antibody and 53BP1 antibody at various time points after RT under normoxia condition (**Figure** **3**a). The density foci in the nuclei of 4T1 cells were markedly elevated after treated with Au@AgBiS_2_‐PEG (Group VI) compared any other groups.

We investigated the kinetics of DNA damage repair at different time points after RT (Figure 3b,e). As shown in Figure 3c,f, the number of DNA damage foci (γ‐H_2_AX and 53BP1) in the Au@AgBiS_2_‐PEG (group VI) slightly decreased by 16.68% and 17.31% at 4 h, and by 33.65% and 29.75% at 24 h post‐irradiation. In contrast, the number of DNA damage foci in the AgBiS_2_ group at an equivalent concentration sharply reduced by 39.05% and 38.21% at 4 h, and by 66.77% and 67.03% at 24 h (Figure S6b,e,c, Supporting Information). Furthermore, the number of DNA damage foci (γ‐H_2_AX and 53BP1) quantified at 24 h after RT was 47.48 ± 3.17 and 48.64 ± 3.50 per cell, respectively (Figure 3d,g), compared with 21.32 ± 3.50 and 19.14 ± 2.25, respectively, per cell for AgBiS_2_ pretreated with RT (Figure S6b,e,d, and g, Supporting Information). These findings suggest that our novel Au@AgBiS_2_‐PEG core‐shell structure exhibits superior efficacy in inducing DNA damage and inhibiting concurrent repair.

To assess the radiosensitivity potential of Au@AgBiS_2_‐PEG as a radiosensitizer, a colony formation analysis was conducted on triple‐negative breast cancer cells (4T1) using different formulations in vitro. Specifically, the number of cell colonies was counted on day 10 after irradiation with various doses to analyze the self‐renewal ability of 4T1 cells. Consistent with the DNA damage assay, the results showed a significant reduction in the cloning efficiency of 4T1 cells treated with Au@AgBiS_2_‐PEG compared with that of the Au NR group (Figure 3h,i). Following RT (at 6 Gy) combined with Au NRs and Au@AgBiS_2_‐PEG, the SF drastically reduced by 31.43% and 62.05%. The SER values in the treatment groups of PBS + RT, Au NRs + RT, and Au@AgBiS_2_‐PEG + RT were 1.0, 1.67, and 2.30, respectively, whereas different concentrations of AgBiS_2_ (25, 50, and 100 µg mL^−1^) had SER values of 1.41, 1.73, and 1.88, respectively (Figure 3j and Figure S7, Supporting Information).

Next, we investigated the underlying mechanism of enhanced radiosensitization by Au@AgBiS_2_‐PEG by detecting cellular ROS generation. After ionizing irradiation, ROS formation plays two crucial roles. First, abundant ROS may attack the covalent bonds of DNA, contributing to cell apoptosis. Additionally, ROS inevitably induce DNA damage and form stable DNA peroxides, inhibiting DNA damage repair. Therefore, we quantified intracellular ROS levels using ROS assay kits and confocal laser scanning microscopy (CLSM) detection (Figure 3k). The results revealed a drastic elevation in intracellular ROS levels following irradiation at 6 Gy. Cells treated with Au@AgBiS_2_‐PEG exhibited 3.14‐fold higher ROS levels. Then the ROS generation levels of Au@AgBiS_2_‐PEG (group VI) increased even further to 1.56‐fold compared with that of Au (group V) due to the effect of AgBiS_2_ after irradiation (Figure 3l). Thus, the intracellular ROS concentration results were consistent with the DNA damage results.

### 2.3 In Vitro Toxicity Evaluation and Detection of Pyroptosis Induced by Au@AgBiS_2_ Under Radiation Conditions

**Figure 4:**
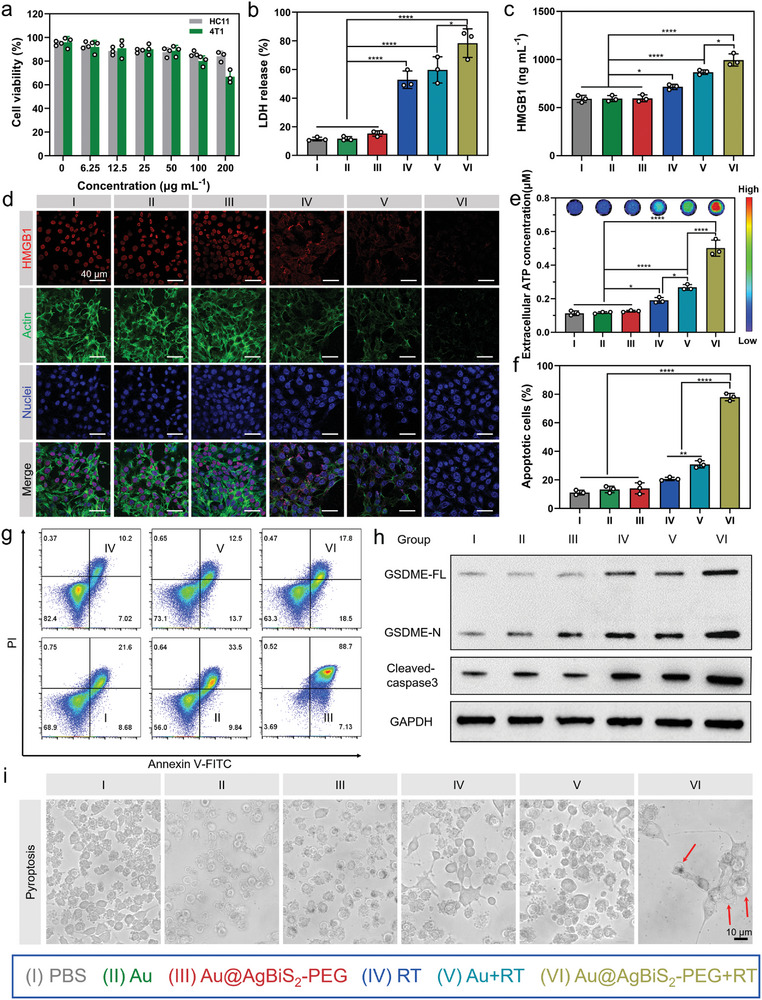
Intracellular cytotoxicity and characterization of the pyroptosis‐inducing performance of Au@AgBiS_2_‐PEG. a) Relative viability of 4T1 and HC11 cells treated with Au@AgBiS_2_‐PEG with respective concentrations. b) Release of LDH in 4T1 cells treated with different formulations (n = 3). c) Quantitative analysis of HMGB1 release in 4T1 cells with respective formulations by ELISA (n = 3). d) Representative immunofluorescence images of release of HMGB1 in 4T1 cells with diverse formulations (n = 3). e) Release of ATP in 4T1 cells treated with different formulations (n = 3). f) Quantitative analysis of the percentage of apoptosis cells after various formulations (n = 3). g) A typical apoptosis assay by using AnnexinV‐FITC and PI co‐staining was detected by Flow‐cytometry. h) Western blot analysis of GSDME‐F and GSDME‐N protein expression in 4T1 cells treated with respective formulations. i) Cell morphology images depicting diverse treatments. Cell swelling (red arrow) observed in the Au@AgBiS_2_‐PEG combined with RT group. Scale bars = 10 µm. Data are indicated as the mean ± SD, and analyzed with one‐way analysis of variance (ANOVA) (*P < 0.05, **P < 0.01, ***P < 0.001, ****P < 0.0001).

In vitro toxicity evaluation of Au@AgBiS_2_‐PEG was conducted on 4T1 cells under irradiation condition or not by using CCK‐8 assay. The results revealed dose‐dependent cytotoxicity on 4T1 cells, with a viability of 66.84% ± 5.38% observed at a concentration of 200 µg mL^−1^ (Figure S8, Supporting Information). Furthermore, the viability of HC11, HUVEC, and 3T3‐NIH cells were assessed using the same method, and the viability of 84.11% ± 4.30%, 75.78% ± 4.02%, and 77.03% ± 2.18% were observed, respectively (**Figure** **4**a; Figure S9, Supporting Information). Based on the remarkable radiosensitization effect of Au@AgBiS_2_‐PEG, we hypothesized that Au@AgBiS_2_‐PEG can induce large amounts of Ag ions release under high energy irradiation conditions, resulting in breaking intracellular homeostasis. Then we detected the Au@AgBiS_2_‐PEG concentration in the supernatant by inductively coupled plasma mass spectrometer (ICP‐MS). The results reflected Ag ion and Bi ion concentration in Au@AgBiS_2_‐PEG treated with 6 Gy irradiation was 1265.31 ± 350.33 µg L^−1^ and 354.811 ± 66.05 µg L^−1^ corresponding to 524.776 ± 120.71 µg L^−1^ and 144.332 ± 32.78 µg L^−1^, with non‐RT treatment respectively (Figure S10, Supporting Information). Therefore, the released Ag from Au@AgBiS_2_‐PEG play an important role in activating immune response.^[^ ^25^ ^]^ We hypothesized that it might trigger the release of damage‐associated molecular patterns (DAMPs) in response to ionizing radiation. We then measured the release of lactate dehydrogenase (LDH) and DAMPs, including ATP. Figure 4b shows that the LDH concentration in 4T1 cells was significantly elevated in the Au@AgBiS_2_‐PEG + RT group compared with that in all other groups, indicating that Au@AgBiS_2_‐PEG, when combined with RT, potentiates pyroptosis in an efficient manner. HMGB1, a nucleus‐localizing high mobility group box 1 protein, is considered a crucial late inflammatory factor and bears greater significance than tumor necrosis factor (TNF) and interleukin‐1 (IL‐1) in the early stages of inflammation. Enzyme‐linked immunosorbent assay (ELISA) showed the concentration of HMGB1 in the supernatant of Au@AgBiS_2_‐PEG + RT was ≈1.39‐fold and 1.14‐fold higher than that in the RT alone and Au NRs + RT groups, respectively (Figure 4c). Laser confocal microscopy revealed that the Au@AgBiS_2_‐PEG + RT treatment resulted in the highest release of HMGB1 into the medium, accompanied by the destruction of tumor cell membranes and cytoskeleton (Figure 4d). Among these DAMPs, surface‐exposed calreticulin (CRT) acts as a potent phagocytic signal by binding to CD91 receptors on phagocytes during the pre‐apoptotic phase.^[^ ^26^ ^]^ The MFI of CRT in the Au@AgBiS_2_‐PEG + RT group was significantly elevated, ≈2.00 times and 1.43 times higher than that in the RT alone and Au NRs + RT, groups respectively (Figure S11, Supporting Information). Extracellular ATP acts as a chemoattractant in the early and middle stages, recruiting dendritic cells (DCs) for maturation and differentiation. Bioluminescence imaging using luciferase‐based ATP probes showed that after 24 h of treatment with Au@AgBiS_2_‐PEG + RT, the amount of extracellular ATP was 2.63 times and 1.87 times higher than that after RT alone and Au NRs + RT, respectively (Figure 4e).

According to previous literature, the release of DAMPs triggered by irradiation can induce pyroptosis.^[^ ^27^ ^]^ To verify our hypothesis, annexin V/propidium iodide (PI) double‐positive cells, indicating membrane integrity loss in pyroptotic cells, were detected. The percentage of these cells in the Au@AgBiS_2_‐PEG + RT group (group VI) was 77.9% ± 2.55%, which was ≈3.99‐fold and 2.67‐fold higher than that in the RT alone and Au NRs combined with RT groups, respectively (Figure 4f,g). Gasdermin family proteins were identified as the key executive molecules to induce pyroptosis. Furthermore, it is crucial to distinguish between the N‐ and C‐terminal domains of GSDME. When GSDME was cleaved by activated caspase 3, it produces the GSDME N‐fragment, which was responsible for membrane perforation and induction of pyroptosis. The level of the GSDME N‐terminal fragment (GSDME‐N) and cleaved caspase‐3 in 4T1 cells treated with various formulations were detected by western blotting to explore the mechanism underlying pyroptosis. The results showed that GSDME in the Au@AgBiS_2_‐PEG group treated with RT was remarkably cleaved and activated by caspase‐3 compared with that in the other treatment groups. The levels of GSDME‐N and cleaved caspase‐3 in the Au@AgBiS_2_‐PEG + RT (group VI) were 3.91‐fold and 3.64‐fold higher than in the PBS group (group I), respectively (Figure 4h; Figure S12, supporting information). To verify the caspase 3 activation induced by Ag^+^ ions release, we first detected the cell uptake of Ag@AgBiS_2_ for different time points for different time points (0, 4, 8, 12 h) to determine the Ag^+^ ions concentration by ICP‐MS. The result showed the amount of Ag^+^ ions release treated by RT were 3.0‐fold than non‐RT treatment (Figure S13, supporting information). Notably, the cleaved caspase 3 and GSDME‐N expression in Ag^+^ ions group exhibited 5.48‐fold and 4.86‐fold higher than in the PBS group, while the level of cleaved caspase 3 and GSDME‐N in the Au@AgBiS_2_ group (group III) was low, indicating that RT might trigger ions release in Au@AgBiS_2_‐PEG, thereby inducing pyroptosis. (Figure S14, supporting information).

Next, inverted microscopy was used to observe the morphology of 4T1 cells in the different treatment groups. As shown in Figure 4i, noticeable cell swelling with big bubbles was observed in the Au@AgBiS_2_‐PEG group treated with RT (group VI), whereas the other groups displayed almost no obvious balloon‐like cells. The process of pyroptosis was observed by detecting 4T1 cells treated with Au@AgBiS_2_‐PEG combined with RT at different time intervals (0–48 h) using a fully automatic live cell imaging system (Figure S15, Supporting Information).

### 2.4 The Biodistribution of Au@AgBiS_2_‐PEG and CT Imaging Performance In Vitro and In Vivo

**Figure 5:**
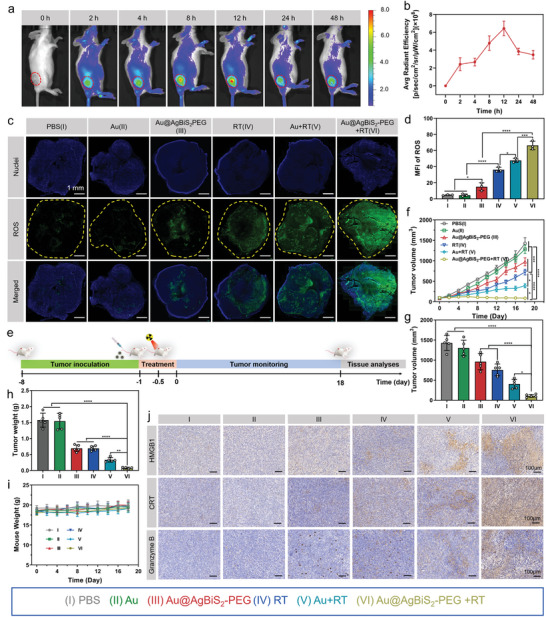
Tumor accumulation study and ROS detection in vivo and performance of antitumor efficacy by Au@AgBiS_2_‐PEG combined with RT. a) Fluorescence images of 4T1 bearing‐tumors mice intravenously injected with Cy5.5‐labeled Au@AgBiS_2_‐PEG at various time points (indicated by red dashed circles). b) Quantifications of tumoral mean fluorescence intensity (MFI) of ROS at the respective time points (n = 3). c) Typical images of ROS in tumor slices post different formulations. d) Quantitative analysis of ROS in tumor slices (n = 3). e) Treatment schedule of mice bearing 4T1 tumors treated by Au@AgBiS_2_‐PEG combined with RT. f) Tumor growth curves of 4T1 bearing‐tumors mice received different formulations (n = 5). g,h) Tumor volume and weight after various treatments. i) The changes of mouse body weight post received various treatments. j) Representative staining images of HMGB1, CRT, and Granzyme B of the tumor tissues. Scale bar is 100 µm (n = 3). Data are indicated as the mean ± SD, and analyzed with one‐way analysis of variance (ANOVA) (*P < 0.05, **P < 0.01, ***P < 0.001, ****P < 0.0001).

The accumulation of nano‐sensitizers and pyroptotic inducers in tumors is crucial for enhancing effective antitumor effects and triggering immune responses. Therefore, we conducted further investigations to examine the distribution and tumor retention of Au@AgBiS_2_‐PEG NPs. We successfully loaded the fluorescent dye Cy5.5 into Au@AgBiS_2_‐PEG, enabling fluorescence imaging and ensuring stability, facilitated by the hydrophobic interaction and van der Waals forces between the dye and NPs. To assess the in vitro biocompatibility of Au@AgBiS_2_‐PEG, a cell uptake assay was performed. Initially, we investigated the cell uptake mechanism of Au@AgBiS_2_‐PEG. As shown in Figure S16 (Supporting Information), 4T1 cells were co‐incubated with Cy5.5‐labeled Au@AgBiS_2_‐PEG NPs. We used phalloidin‐488 to visualize the cytoskeleton and quantified the intracellular MFI at different time intervals (0‐48 h) after incubation. The Au@AgBiS_2_‐PEG content within the cells increased as the cellular uptake time increased, reaching its peak at 4 h post‐incubation. Subsequently, we examined the in vivo distribution of Cy5.5‐labeled Au@AgBiS_2_‐PEG NPs after intravenous injection. As depicted in **Figure** **5**a, the fluorescence intensity of Cy5.5‐labeled Au@AgBiS_2_‐PEG gradually increased and was observed in 4T1 tumor tissues. This finding demonstrated the remarkable ability of Au@AgBiS_2_‐PEG NPs to accumulate in tumors. The fluorescence density of Cy5.5 steadily increased and reached its peak intensity within the first 12 h, followed by a gradual decay owing to metabolism, indicating the excellent tumor accumulation capability of Au@AgBiS_2_‐PEG NPs (Figure 5a,b). Furthermore, major organs were collected and subjected to bioluminescent imaging analysis at 48 h after injection (Figure S17a, Supporting Information). The fluorescence intensity was primarily detected in the liver and tumor sites. Specifically, the fluorescence intensity at the tumor site was 2.79‐fold and 8.12‐fold higher than that in the kidney and spleen, respectively (Figure S17b, Supporting Information). These results were consistent with the previous fluorescence imaging assays, confirming the preferential accumulation of Au@AgBiS_2_‐PEG nanoparticles in the major organs and tumors. Based on these imaging and metabolism results in vivo, we recommend a 12‐h post‐injection time point for irradiation treatment as the optimal time window.

The distribution of Ag ions and Bi ions in vital organs, including the tumor, as well as their metabolites in urine and feces, was analyzed at different time points (0, 1, 7, and 15 days) using ICP‐MS detection (Figure S18, Supporting Information). Additionally, the routine blood detection and blood biochemical analysis were assessed by enzyme linked immunosorbent assay (Elisa) in the Au@AgBiS_2_‐PEG injection group at diverse time points (0, 1, 7, and 15 days). The results showed no obvious abnormalities were observed in vital organs (Figure S19, Supporting Information). Meanwhile, the major organs also were collected by HE staining at various time points (0, 1, 7, and 15 days) and no significant abnormalities were observed in the cellular morphology (Figure S20, Supporting Information). Considering the high atomic number of elemental Bi and the presence of doped elemental Au, we hypothesized that Au@AgBiS_2_‐PEG NPs could exhibit excellent CT imaging performance because of their superior X‐ray attenuation coefficients. In vitro CT images and corresponding CT values of Au@AgBiS_2_‐PEG NPs were obtained to evaluate their CT imaging capability. As shown in Figure S21 (Supporting Information), the Hounsfield Unit (HU) value increased linearly with the concentration of Au@AgBiS_2_‐PEG NPs. Specifically, at a concentration of 10 mg mL^−1^, the CT value of Au@AgBiS_2_‐PEG reached 294.39 HU, whereas the clinical contrast agent iohexol exhibited a CT value of 261.41 HU at an equivalent concentration. Subsequently, we investigated the feasibility of using Au@AgBiS_2_‐PEG NPs as contrast agents for in vivo CT imaging through intravenous injection. Au@AgBiS_2_‐PEG solutions (10 mg kg^−1^) were injected into mice bearing 4T1 tumors, and CT images were acquired at 0, 4, 8, 12, and 24 h after injection, with iohexol used as a reference. As shown in Figure S22a,b (Supporting Information), the Au@AgBiS_2_‐PEG NPs produced time‐dependent contrast enhancement at the tumor site, with the optimal CT signal observed at ≈12 h post‐injection. In contrast, the contrast enhancement signal from iohexol was barely detected at 4 h post‐injection. Notably, the contrast signal from Au@AgBiS_2_‐PEG remained detectable even 24 h after injection, indicating excellent accumulation of the NPs in the tumor region through the enhanced permeability and retention effect. Moreover, CT contrast enhancement signals were observed in the heart and liver at 4 and 8 h post‐intravenous injection (Figure S22c, Supporting Information). Notably, no significant CT contrast signal was detected in the heart and liver at 24 h after injection of the Au@AgBiS_2_‐PEG solution, suggesting effective elimination of the NPs without causing systemic toxicity.

### 2.5 ROS Detection In Vivo and Effective Antitumor Effect by Au@AgBiS_2_‐PEG Combined with RT

We further detected ROS generation in vivo. DCFH‐DA, a visualization indicator for ROS, was injected intraperitoneally 30 min before RT. It is noteworthy that the Au@AgBiS_2_‐PEG ‐treated tumor alone exhibited weak ROS signals, whereas the PBS group showed increased ROS levels after RT treatment. Tumor ROS signals were considerably elevated from 47.68% ± 2.60% for the Au NRs + RT group to 66.27% ± 5.26% for the Au@AgBiS_2_‐PEG ‐treated with RT group, indicating an enhanced sensitization effect of RT in tumors (Figure 5c,d). Next, the antitumor efficacy of Au@AgBiS_2_‐PEG was evaluated in vivo. Mice bearing 4T1 tumors were pretreated with PBS, Au NRs, or Au@AgBiS_2_‐PEG via intravenous injection, followed by local irradiation with a single fraction of 6 Gy X‐ray to the tumors (Figure 5e). To ensure precise irradiation dose delivery to the tumor tissue, quality assurance (QA) was performed using a 3D dosimetry monitor system in a water phantom.^[^ ^24^ ^]^ The QA measurement confirmed that the maximum dose depth was ≈1.6 cm underwater (Figure S23, Supporting Information). Based on these dosimetry characteristics, the surface of 4T1 tumors in BALB/c mice was covered with a 1‐cm solid water film (bolus) to increase the surface irradiation dose deposition and enhance the efficacy of irradiation while minimizing damage to adjacent tissues surrounding the tumor. Tumor volumes were monitored after different treatments and are shown in Figure 5f. The Au NRs treatment group (group II) did not show significant inhibition of tumor growth compared with the PBS group (group I). In contrast, Au@AgBiS_2_‐PEG alone (group III) exhibited moderate tumor inhibition effects (32.79% inhibition of tumor growth), and RT alone (group IV) showed a similar trend (47.11% inhibition of tumor growth). Importantly, a significant delay in tumor growth was observed in the Au NRs + RT treatment group, with a 71.66% inhibition of tumor growth (group V). Furthermore, the strongest inhibition effect was observed when Au@AgBiS_2_‐PEG was combined with irradiation, with a tumor growth inhibition rate of 92.29% (group VI). This finding illustrates the remarkable ROS production of Au@AgBiS_2_‐PEG triggered by RT, along with increased DNA damage and inhibition of repair (Figure 5g). Measurement of excised tumor weight confirmed that the therapeutic efficacy on tumors was significantly enhanced in the Au@AgBiS_2_‐PEG +RT group (group VI) (Figure 5h). No significant weight fluctuations were observed in any of the treatment groups (Figure 5i). Major organs were collected and subjected to H&E staining on the 18th day post‐treatment, and the results showed no significant systemic toxicity, demonstrating the excellent biosafety of the treatment (Figure S24, Supporting Information). Furthermore, the antitumor effect of AgBiS_2_ was evaluated at different concentrations. On the 18th day post‐treatment, AgBiS_2_ (10 mg kg^−1^) combined with RT at 6 Gy (group VI) exhibited a 68.8% inhibitory effect on tumor growth, whereas AgBiS_2_ (5 mg kg^−1^) combined with RT at 6 Gy (group V) showed a moderate inhibitory effect on tumor growth (54.6%). Only when the radiation dose was increased, the tumor inhibition rate of AgBiS_2_ (10 mg kg^−1^) combined with RT at 8 Gy (group VI) reached 87.63%. However, it should be noted that higher radiation doses may cause irreversible adverse effects on normal tissues. H&E staining images of whole tumors reflected that AgBiS_2_ (10 mg kg^−1^) combined with RT at 8 Gy (group VII) treatment produced moderate necrotic cells compared with AgBiS_2_ (10 mg kg^−1^) combined with RT at 6 Gy (group VI), which could be attributed to the effect of high‐dose irradiation (Figure S25, Supporting Information).

To evaluate the antitumor effects of Au@AgBiS_2_‐PEG combined with RT, the tumors were dissected for analysis of the cellular proliferation antigen Ki‐67 and TUNEL staining, which showed the Au@AgBiS_2_‐PEG +RT (Group VI) caused significant damage to tumor cells, and Au NRs treated by RT (Group V) displayed the moderate levels of tumor cells necrosis and apoptosis (Figure S26, Supporting Information). In addition, we also confirmed whether Au@AgBiS_2_‐PEG treated by RT‐induced pyroptosis via enough DAMPs release in vivo. The DAMPs including HMGB1 and CRT were detected by using immunohistochemical staining. No significant CRT exposure or HMGB1 release was observed between the Au@AgBiS_2_‐PEG nanoparticles alone treatment group and the PBS group. The elevated expression of CRT and HMGB1 in the tumors were detected by Au@AgBiS_2_‐PEG nanoparticles combined irradiation group, which could drive the emission of DAMPs in vivo and further contribute to an effective immune response against residual tumor cells (Figure 5j). Granzyme B as the primary effector molecule can enter the target cells and activate the caspase cascade, thus rapidly causing the DNA break of the target cells and leading to rapid apoptosis.^[^ ^28^ ^]^ Then tumors with various treatments were removed to detect the granzyme B expression by immunohistochemistry (IHC) staining. The elevated level of granzyme B was observed in Au@AgBiS_2_‐PEG +RT (Group VI) (Figure 5j). Collectively, the enhanced synergistic RT by Au@AgBiS_2_‐PEG (Group VI) mainly attributed to three aspects: (1) the high‐Z element radiosensitization effect via Bi and Au NRs to deposit more radiant energy focused on the tumor; (2) the dramatical ROS production of Au@AgBiS_2_‐PEG triggered by RT could kill more tumor cells; (3) AgBiS_2_ can obviously induce cancer cells‐specific cytotoxicity in TME. Collectively, these factors contribute to the enhanced antitumor efficacy observed with the combination treatment.

### 2.6 In Vivo Pyroptosis Induction of Au@AgBiS_2_‐PEG Treated by RT to Promote the Antitumor Immune

**Figure 6:**
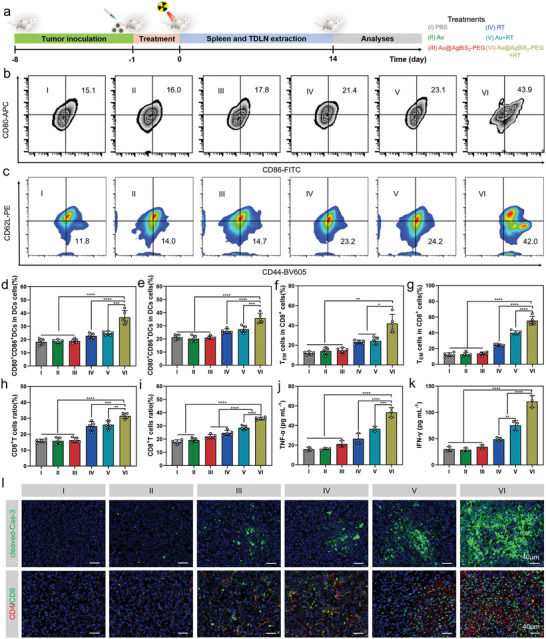
The Au@AgBiS_2_ combined with RT exhibited an excellent antitumor response in 4T1 tumor models. a) Treatment schedule of 4T1 bearing‐tumor mice treated with various formulations. b,c) Representative flow cytometry plots and analysis of the proportions of mature DCs and effector memory (EM) cells in splenocytes. d,e) Quantitative analysis of the percentages of mature DCs in the splenocytes and TDLN. The proportions of effector memory (EM) cells in CD8^+^ cells f,g) and CD8^+^ T cells h,i) in splenocytes and TDLN of mice, respectively. j,k) The concentration of relative cytokines (TNF‐α, IFN‐γ) of serum post various treatments. l) Immunofluorescence staining of cleaved caspase‐3 (c‐Cas‐3) and colocalization of CD4 and CD8 in tumors (n = 3). Data are indicated as the mean ± SD, and analyzed with one‐way analysis of variance (ANOVA) (*P < 0.05, **P < 0.01, ***P < 0.001, ****P < 0.0001).

The ability of Au@AgBiS_2_‐PEG to induce pyroptosis under X‐ray irradiation prompted us to investigate its immune response in vivo. When the tumor volumes reached ≈100 mm^3^, 4T1 tumor‐bearing mice were divided into six groups: (I) PBS, (II) Au NRs, (III) Au@AgBiS_2_‐PEG, (IV) RT, (V) Au NRs + RT, and (VI) Au@AgBiS_2_‐PEG + RT. Groups II, III, V, and VI were intravenously injected with Au NRs or Au@AgBiS_2_‐PEG (10 mg kg^−1^). After 12 h, mice in groups IV, V, and VI were exposed to a single fraction of 6 Gy X‐ray. As shown in **Figure** **6**a, the 4T1 tumor‐bearing mice were sacrificed on day 22 after different treatments. To investigate the effects induced by the pyroptosis of Au@AgBiS_2_‐PEG in combination with RT in the tumor immune microenvironment (TIME), we further analyzed the changes in immune cell populations within the TIME.

CD4^+^T cells not only have direct toxic side effects on tumor cells, but also have auxiliary effects on the activation and proliferation of CD8^+^T cells, helping to generate and ensure long‐term memory based cytotoxic T lymphocyte (CTL) responses. The killing effect of CD4^+^T cells on tumors is mainly achieved through IFN‐γ dependency mediated by mechanisms. We first observed the percentage of IFN‐γ^+^ CD4^+^ T cells immune cells in tumor. The results showed the number of IFN‐γ^+^ CD4^+^ T cells in tumor was 2.35‐fold and 1.56‐fold higher in the Au@AgBiS_2_‐PEG +RT group than that in the RT group and Au + RT group, respectively (Figures S27, and S28a,c, Supporting Information). Furthermore, it has been reported that CD3^−^CD49b^+^ cells were regarded as the infiltrating NK cells to elicit immunogenic tumor microenvironment.^[^ ^29^ ^]^ In the Au@AgBiS_2_‐PEG combined with RT group, the CD45^+^CD3^−^CD49b^+^ (NK cells) levels in tumor were 1.72‐fold and 1.28‐fold higher than that in RT alone group and Au + RT group, respectively (Figure S28b,d, Supporting Information).

Then we detected the immune cells from the spleen and tumor‐draining lymph nodes (TDLNs), and single‐cell suspensions were subjected to flow cytometry analysis. DC maturation is crucial for antigen‐specific T cell activation and enhancement of antitumor immunity. Therefore, mature DCs (CD80^+^ CD86^+^ in CD11c^+^ cells) were quantified in the spleen and TDLNs. The number of mature DCs in splenocytes and TDLNs was 2.01‐fold and 1.77‐fold higher in the Au@AgBiS_2_‐PEG + RT group than in the PBS group, respectively (Figure 6b,d,e; Figures S29 and S30, Supporting Information). In the Au@AgBiS_2_‐PEG combined with RT group, the number of CD8^+^ T cells was significantly higher than in any of the other groups. In particular, CD8^+^ T cell levels in splenocytes and TDLNs were 1.98‐fold and 2.0‐fold higher than those in the PBS group, respectively (Figure 6h,I, Figures S32a and S33a, Supporting Information), indicating that Au@AgBiS_2_‐PEG treated with RT effectively promoted T cell activation and DC maturation. These results demonstrate that pyroptosis induced by Au@AgBiS_2_‐PEG in combination with RT can trigger a potent antitumor immune response owing to its unique cell death pattern, which is associated with the release of inflammatory molecules and cellular contents.

To explore whether Au@AgBiS_2_‐PEG + RT treatment could potentiate the immune memory effect, the expression of CD44 and CD62L surface markers on T cell subsets was evaluated to detect the proportion of effector T cells (T_EM_) in splenocytes and TDLNs, which provide long‐term immune protection. An evident enhancement trend of antigen‐stimulated T cells (T_EM_, CD3^+^CD4^+^CD44^+^CD62L^−^) in the spleen and TDLNs was observed in the Au@AgBiS_2_‐PEG + RT group (group VI) (Figure 6c, Figures S31, S32, and S33 in Supporting Information). In particular, the percentage of TEM cells in CD4^+^ and CD8^+^ cells of the spleen and TDLNs post‐treatment with Au@AgBiS_2_‐PEG + RT was 3.64‐fold, 2.97‐fold, 4.54‐fold, and 3.56‐fold higher than that of the PBS group, respectively (Figure 6f,g, Figure S32b, S33b,c, S34 in Supporting Information). In addition, TNF‐α and IFN‐γ are two key inflammatory immune cytokines in the TME. TNF‐α modulates immune activity and promotes the death of tumor cells by T cells and other killer immune cells, whereas IFN‐γ inhibits tumor growth by activating macrophages, upregulating antigen processing, and increasing the production of presentation molecules. The levels of TNF‐α and IFN‐γ were evaluated in the TME using ELISA. The results showed that the levels of TNF‐α and IFN‐γ were considerably elevated in the Au@AgBiS_2_‐PEG + RT group. Specifically, the concentration of TNF‐α and IFN‐γ in the serum of 4T1 tumor‐bearing mice pretreated with Au@AgBiS_2_‐PEG combined with RT was 3.36‐fold and 4.03‐fold higher than that in the PBS group, respectively (Figure 6j,k).

Similar results were verified in tumor tissues using immunofluorescence staining, where a significant fluorescence density of CD4/CD8 colocalization and cleaved caspase‐3 was detected in the Au@AgBiS_2_‐PEG + RT (group VI), which is crucial for pyroptosis induction and cytotoxic T cell‐mediated immune therapy (Figure 6l). Thus, we conclude that the release of DAMPs triggered by Au@AgBiS_2_‐PEG in combination with RT induced pyroptosis, which could effectively potentiate the activation of immune cells, particularly cytotoxic T cells and prime adaptive immune responses. Furthermore, TNF‐α and IFN‐γ are two key inflammatory immune cytokines in the TME. TNF‐α modulates immune activity and promotes the death of tumor cells by T cells and other killer immune cells, whereas IFN‐γ inhibits tumor growth by activating macrophages, upregulating antigen processing, and increasing the production of presentation molecules. The levels of TNF‐α and IFN‐γ were evaluated in the TME using ELISA. The results showed that the levels of TNF‐α and IFN‐γ were significantly elevated in the Au@AgBiS_2_ + RT group. Specifically, the concentration of TNF‐α and IFN‐γ in the serum of 4T1 tumor‐bearing mice pretreated with Au@AgBiS_2_ combined with RT was 3.36‐fold and 4.03‐fold higher than that in the PBS group, respectively (Figure 6j,k).

### 2.7 Tumor Metastasis Prevention by Au@AgBiS_2_‐PEG _+_ Radiotherapy Based on Subcutaneous and Metastatic 4T1 Tumor Model

**Figure 7:**
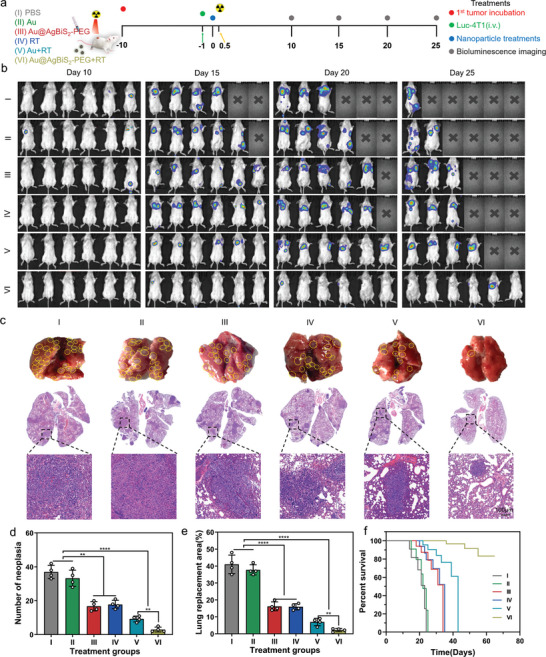
Au@AgBiS_2_‐PEG combined with RT‐induced pyroptosis for prevention of tumor metastasis. a) The treatment schedule for mice with different formulations to inhibit tumor metastasis. b) In vivo representative bioluminescence images showing metastatic nodules and the tracking of the spread and growth of intravenously injected 4T1‐luciferase tumor cells in mice on day 10, 15, 20, and 25 according to the treatment schedule. (n = 6). c) Representative photographs of metastatic lung nodules (top) with yellow dashed circles indicating metastatic nodules, and corresponding H&E staining images of metastatic lung nodules (middle and bottom). Scale bar = 100 µm. d) Quantification of metastatic lung nodules after different treatments. (n = 4). e) The proportion of the lung replacement area observed after treatments with various formulations (n = 4). f) Survival analysis of mice treated with different formulations (n = 6). Data are indicated as the mean ± SD, and analyzed with one‐way analysis of variance (ANOVA) (*P < 0.05, **P < 0.01, ***P < 0.001, ****P < 0.0001).

Encouraged by the efficacy of Au@AgBiS_2_‐PEG combined with radiotherapy in the primary tumor, the established protective anti‐tumor immune response and the systemic anti‐tumor memory, we designed an experiment to evaluate the efficacy of Au@AgBiS_2_‐PEG combined with radiotherapy in the prevention of lung metastasis. To establish an aggressive metastasis mode, we first designed the breast cancer subcutaneous tumor model by 4T1 cells injection. As shown the **Figure** **7**a, when tumor volumes reached 100 mm^3^, the stable expression luciferase 4T1 (luc‐4T1) cells were intravenously injected into the 4T1 bearing‐tumor mice on day 9. The primary tumor was received different treatments after 24 h. In vivo the bioluminescence images of mice were detected by the IVIS imaging system at 10, 15, 20, and 25 days after intravenous injection of Luc‐4T1 cells. Notably, increasing bioluminescent signals as an indicator of tumor metastasis were captured in tumor‐bearing mice treated only with PBS, Au NRs, and Au@AgBiS_2_‐PEG even after 10 days of intravenous injection with Luc‐4T1 cell. The Au@AgBiS_2_‐PEG in combination with RT exhibited a stronger inhibition signal of tumor metastasis compared with RT alone and Au+RT (Figure 7b). It suggested that the process of tumor metastasis prevention was attributed to the drastic immune response of the Au@AgBiS_2_‐PEG combined with RT mediated‐pytoptosis. Next, we further evaluated the lung metastasis by the representative photographs and H&E staining of the whole lung via counting the number of tumor nodules in every lung lobe (Figure 7c). Compared to PBS group, the number of metastatic nodules on the lung and the proportion of lung replacement area (from photographs) and that of new tumor nodules on the lung lobes (from H&E images) after Au@AgBiS_2_‐PEG +RT treatment were significantly reduced (Figure 7d,e). In this process, the survival analysis showed about 50% of mice pretreated with Au@AgBiS_2_‐PEG +RT could survive 60 days in Au@AgBiS_2_‐PEG +RT group, while mice received the other treatments all died within 14 to 43 days (Figure 7f). In conclusion, our results suggest that Au@AgBiS_2_‐PEG nanoparticles combined with RT have an excellent inhibitory effect on the growth and metastasis of primary tumors, accompanied by long‐term survival.

## 3 Conclusion

Our study presents a rational design of the core‐shell structure of Au@AgBiS_2_‐PEG using an intermediate layer conversion method. The resulting Au@AgBiS_2_‐PEG core‐shell structure exhibits enhanced DNA damage under irradiation conditions owing to the presence of high‐Z elements. Additionally, Ag ion release from Au@AgBiS_2_‐PEG promotes charge separation, leading to increased ROS generation under irradiation and enhances DNA damage caused by RT, thereby sensitizing cancer cells to RT. We have also demonstrated that Au@AgBiS_2_‐PEG, when triggered by irradiation, induces pyroptosis through GSDME cleavage by activated caspase‐3 and releases DAMPs in vitro. In vivo, the combination of Au@AgBiS_2_‐PEG and RT promotes significant ROS production, triggering a robust antitumor immune response and effectively preventing lung and systemic metastasis, highlighting the potent pyroptosis induction ability of Au@AgBiS_2_‐PEG. Furthermore, our tumor therapeutic experiments have confirmed the excellent biocompatibility and biosafety of Au@AgBiS_2_‐PEG, as well as its remarkable ability to inhibit proliferation and induce apoptosis. This work not only provides a novel strategy for fabricating metal‐semiconductor hybrids using our synthetic methods but also offers insights and inspiration for radiosensitization and RT‐triggered pyroptosis‐mediated cancer immunotherapy.

## 4 Experimental Section

### Materials and Instruments

Hexadecyltrimethylammonium bromide (CTAB, 99.9 wt%) was purchased from Shanghai Macklin Biochemical Co., Ltd. Silver nitrate (AgNO_3_, 99.8%) was purchased from Shanghai Aladdin Biochemical Technology Co., Ltd. Tetrachloroauric acid tetrahydrate (HAuCl_4_·4H_2_O, AR), Zinc nitrate hexahydrate (Zn(NO_3_)_2_·6H_2_O, 99.0 wt%), Bismuth nitrate pentahydrate (Bi(NO_3_)_3_·5H_2_O, 99.0 wt%), 1‐ascorbic acid (AA, 99.7 wt%), ethylene glycol ((CH_2_OH)_2_, 99.0%), thiourea (H_2_NCSNH_2_, 99.0 wt%), methanol (CH_3_OH, 99.5 wt%), and hexamethylenetetramine (HMTA, 99.0 wt%) were purchased from Sinopharm Chemical Reagent Co., Ltd. Poly (maleic anhydride‐alt‐1‐octadecene) (PEG‐C_18_PMH) was purchased from Sigma‐Aldrich Co., Ltd (St Louis, MO, USA). Cell Counting Kit‐8 (CCK8) was purchased from Dojindo Laboratories (Japan). Water was purified by using a Milli‐Q Water System (Mililpore, Bedford, MA) consisting of a carbon filter cartridge, two ion‐exchange filter cartridges and an organic removal cartridge. 4% Paraformaldehyde fix solution were purchased from Biosharp (Beijing, China). Actin‐Tracker Green‐488 and ROS detection were purchased from Beyotime (Shanghai, China). 4, 6‐diamidino‐2‐phenylindol) (DAPI) and Dimethyl sulfoxide (DMSO) were purchased from Sigma–Aldrich (St. Louis, MO). Percoll solution was obtained from GE Healthcare (USA). Anti‐CD4 antibody and anti‐CD4 antibody for immunofluorescence were acquired from Abcam (Abcam, Inc., MA, USA). Anti‐IFN‐γ ELISA kit, anti‐ IL‐12 ELISA kit, and anti‐ TNF‐α ELISA kit were purchased from Dakewe Biotech (Beijing, China). Anti‐ HMGB1 antibody, anti‐Calreticulin antibody, and anti‐Granzyme B antibody were purchased from Abcam (Abcam, Inc., MA, USA). Proliferating cell nuclear antigen Ki‐67 monoclonal antibody (Santa Cruz Biotechnology, Dallas, TX) and TUNEL (Roche Diagnostics, Indianapolis, IN) were analyzed for immunohistochemistry. Anti‐IL‐1β, anti‐NLRP3, anti‐DFNA5/GSDME, and anti‐Cleaved Caspase‐3 antibody were purchased from Abcam (Abcam, Inc., MA, USA). Anti IL‐18 and ACS1 were obtained from Bioworld (Bloomington, MN 55 425, USA) and Bioss company (Boston, USA), respectively. GAPDH was purchased from Zsbio company (Beijing, China). Anti‐γ‐H_2_AX mouse monoclonal antibody (Sigma–Aldich, USA), anti‐53BP1 mouse monoclonal antibody (Abcam Inc., MA, USA), anti‐mouse Cy3 and FITC secondary antibody were detected for DNA damage assay. FITC anti‐mouse CD3ε, APC anti‐mouse CD4, APC/Cyanine7 anti‐mouse CD8a, Brilliant Violet 605™ anti‐mouse/human CD44, PE anti‐mouse CD62L, PE/Cyanine7 anti‐mouse CD11c, FITC anti‐mouse CD80, APC anti‐mouse CD86; all antibodies were purchased from BioLegend (BioLegend, Inc., USA).

The surface morphologies of the products were investigated using transmission electron microscopy (TEM), energy‐dispersive X‐ray (EDX) and high‐resolution transmission electron microscopy (HRTEM) using a JEM‐2100F (JEOL, Japan). X‐ray photoelectron spectra (XPS) were performed on an ESCALAB250Xi X‐ray photoelectron spectrometer (Thermo Scientific, American). Powder X‐ray diffraction (XRD) patterns was carried on an X'Pert PRO MPD X‐ray diffractometer (PANalytical B.V., Holland) equipped with graphite monochromated Cu Kα radiation. UV–vis absorption spectra were obtained by using a Hitachi U‐5100 spectrophotometer (Hitachi High‐Technology Corporation, Japan). Dynamic light scattering (DLS) and Zeta potential measurements were carried out on a Malvern Zetasizer Nano ZS90.

### Cell Culture

Murine breast cancer 4T1 cells were obtained from American Type Culture Collection (ATCC) and cultured at 37 °C in a humidified atmosphere containing 5% CO_2_ (Eppendorf^®^, CellXpert^®^ C170, Germany). The cells were cultured in normal RPMI 1640 culture medium (Wisent®, Canada) supplemented with 10% fetal bovine serum (FBS, ExCell Bio, Shanghai, China) and 1% penicillin/streptomycin (Gibco^®^).

### Animals and Tumor Model

Female Balb/c mice at 6–8 weeks of age were obtained from Vital River Laboratories (Beijing, China). All animals were cared in accordance with the guidelines in the Guide to the Care and Use of Experimental Animals, and all procedures were approved by the Animal Care and Use Committee of Anhui Medical University. 1 × 10^6^ 4T1 cells (100 µL) were injected to the right side of Balb/c mice to form a xenograft tumor model.

### Ethics Approval statement

All animal experiments were approved by the Ethics Committee of Anhui Medical University (approval number: LLSC20210077). All animal experiments were performed in accordance with the guidelines of the Association of Laboratory Animal Sciences and the Center for Laboratory Animal Sciences at Anhui Medical University.

### Statistical Analysis

Data were indicated as the mean ± SD. The experimental data and differences were analyzed by using GraphPad Prism 9.3 software (GraphPad, San Diego, CA) with one‐way analysis of variance (ANOVA) with Tukey's multiple comparisons test. Significant difference among group were defined as *p<0.05, **p<0.01 and ***p<0.001, ****P < 0.0001, respectively. P<0.05 was considered to have a significant difference (95% confidence level).

## Conflict of Interest

The authors declare no conflict of interest.

## Supporting information

Supporting Information

## Data Availability

The data that support the findings of this study are available on request from the corresponding author. The data are not publicly available due to privacy or ethical restrictions.
