# Supplementary material for: Multifunctional Au@AgBiS2 Nanoparticles as High‐Efficiency Radiosensitizers to Induce Pyroptosis for Cancer Radioimmunotherapy
Source: Adv Sci (Weinh). 2023 Sep 8;10(30):2302141. doi: 10.1002/advs.202302141 (PMC10602534; doi:10.1002/advs.202302141)
Supplement: Supplementary file 1 — Supporting Information [file ADVS-10-2302141-s001.pdf]

ADVANCED SCIENCE
Open Access

Supporting Information

for *Adv. Sci.*, DOI 10.1002/advs.202302141

Multifunctional Au@AgBiS_2 Nanoparticles as High-Efficiency Radiosensitizers to Induce Pyroptosis for Cancer Radioimmunotherapy

Liang Xiao, Benjin Chen, Wanni Wang, Tian Tian, Haisheng Qian, Xiaohu Li* and Yongqiang Yu**

Supporting Information

Multifunctional $Au@AgBiS_2$ Nanoparticles as High-efficiency Radiosensitizers to Induce Pyroptosis for Cancer Radioimmunotherapy

Liang Xiao,[#] Benjin Chen,[#] Wanni Wang,[#] Tian Tian, Haisheng Qian,[*] Xiaohu Li,[*] Yongqiang Yu[*]

Dr. Liang Xiao, Prof. Xiaohu Li, Prof. Yongqiang Yu
Department of Radiology, Research Center of Clinical Medical Imaging, Anhui Province Clinical Image Quality Control Center, The First Affiliated Hospital of Anhui Medical University, Hefei, China, No. 218, Jixi Road, Shushan District, 230022, Hefei, China
E-mail: lixiaohu@ahmu.edu.cn
E-mail: yuyongqiang@ahmu.edu.cn

Dr. Benjin Chen
Department of Pharmacology, School of Basic Medical Sciences,
Anhui Medical University
Hefei, 230032, P. R. China.

Dr. Wanni Wang, Prof. Haisheng Qian
School of Biomedical Engineering, Anhui Provincial Institute of Translational Medicine, Anhui Engineering Research Center for Medical Micro-Nano Devices
Anhui Medical University
Hefei, 230011, P. R. China.
E-mail: shqian@ahmu.edu.cn

Dr. Tian Tian
Department of Oncology, The First Affiliated Hospital of Anhui Medical University, Hefei, Anhui 230036, P. R. China

[#]These authors contributed equally to this work.

Experimental Section

Synthesis and modification of Au@AgBiS$_2$-PEG NPs

Au@AgBiS$_2$ nanospheres were obtained according to a modified protocol. Firstly, Au@Zn[(OH)$_4$]$^{2-}$ NPs were synthesized according to a protocol from our group.[1] Then they were converted to Au@ZnS NPs *via* a gaseous sulfidation reaction. Au@AgBiS$_2$ NPs were prepared by a rapid ions exchange reaction using the as-obtained Au@ZnS NPs, Bi(NO$_3$)$_3$ and AgNO$_3$. Typically, 0.54 mmol thiourea, 160 mg Au@ZnS NPs were added into 40 mL of ethylene glycol (EG) to form suspension solution, which was heated to 130°C with magnetic stirring and kept for 10 min. Subsequently, 60 mL EG solution containing 1.08 mmol Bi(NO$_3$)$_3$·5H$_2$O and 1.08 mmol AgNO$_3$ was rapidly added into the mixture solution and kept at 130°C for 20 min. Finally, the product in black color was washed, collected, and dried for long-term storage.

To synthesis biocompatible ceria nanoparticles, Au@AgBiS$_2$ NPs were coated with PEG-C$_{18}$PMH.[2] Briefly, PEG-C$_{18}$PMH (50 mg) dissolved in DMSO (2.0 mL) was mixed with DMSO containing Au@AgBiS$_2$ NPs (10 mg). The mixture was added into 10 mL of deionized water under ultrasound and kept for 1 h at roon temperature. The excess PEG-C$_{18}$PMH was removed by dialysis against a 10 kDa molecular weight cut-off bag filter. Purified Au@AgBiS$_2$-PEG were stored at 4°C. For the purpose of comparative experiment, AgBiS$_2$ was synthesized using the same method except for the addition of Au nanorods.

Additionally, PEG-C18PMH-Cy5.5 was prepared by replacing amine-terminated poly(ethylene glycol) methyl ethers (mPEG-NH$_2$) with amine-terminated Cy5.5 at a certain molar amount during the production of PEG-C18PMH, and the other steps remained the same.

Cell viability assay in vitro

4T1 cells were seeded into 96-well plates at the density of 6000 cells per well (100 μL of RPMI 1640 medium). Then the cells were incubated with Au@AgBiS$_2$-PEG NPs for 24 h with various concentrations were added and the final concentrations in RPMI 1640 medium are 0,

12.5, 25, 50, 100, 200 μg mL^{-1}, respectively; In the control group, Equal concentration of DMSO was added. The cell viability was calculated using a cell counting kit-8 (CCK-8) assay following the manufacture's protocol. To further evaluate the toxicity effects of Au@$AgBiS_2$-PEG NPs on normal cells, LC11 cells were seeded into 96-well plates at the density of 6000 cells per well (100 μL of RPMI 1640 medium). Then the cells were incubated with Au@$AgBiS_2$-PEG NPs for 24 h with various concentrations (Au@$AgBiS_2$-PEG NPs: 0, 12.5, 25, 50, 100, 200 μg mL^{-1}). The cell viability was calculated using a cell counting kit-8 (CCK-8) assay.

Detection of ROS In Vitro

4T1 cells were seeded on coverslips in a 24-well plate at a density of 5×10^4 cells per well overnight and. After different treatments, the cells were washed three times with PBS. ROS positive and cell nuclei were stained by DCFH-DA (green) and DAPI (blue), respectively, according to the manufacturer's instructions and then visualized by CLSM. The cells were incubated with a fluorescent probe, dichlorodihydrofluorescein diacetate (DCFH-DA, Beyotime Biotechnology, Shanghai, China, 10 μM) for 30 min in dark (37°C).

Colony Formation Assay

For detecting radiosensitization of $AgBiS_2$, 4T1 cells were cultured in 6-well plates at a density of 800 per well for overnight. Cells were then exposed to fresh medium containing different $AgBiS_2$, concentration (0, 25, 50, 100 μg mL^{-1}) for 4 h, then the medium was replaced with fresh culture media followed receiving irradiation at various doses of 0, 2, 4, 6, and 8 Gy, respectively. After different treatments, the cells were washed twice with PBS, and then incubated for an additional 10 days. Colonies were stained with 1.0% crystal violet and counted to evaluate the effects of respective treatments. Data fitting was carried out according to the single-hit multitarget mode: $SF = 1 - (1 - e^{D/D0})$ N (SF, cell survival fraction; D, radiation dose; e, the bottom of the natural logarithm; D0, the mean death dose; N, extrapolate number), Each group was measured in triplicate. For detecting radiosensitivity effects of Au@$AgBiS_2$: 4T1

cells were divided into five groups: (I) PBS, (II) Au, (III) Au@$AgBiS_2$, (IV) RT, (V) Au+RT, (VI) Au@$AgBiS_2$-PEG+RT at an equivalent Au@$AgBiS_2$-PEG concentration of 100 μg mL^{-1}. Other experimental methods are consistent with the above.[3]

Calculation of Sensitization Enhancement Ratio (SER)

The cell survival fraction of each group was calculated by the ratio of the number of colony formed by seeded cells after various treatments versus the number of colonies formed by untreated cells. The classical multitarget single-hit model was applied to perform nonlinear fitting for the cell survival fraction of each group using Graphpad prism 9 software (GraphPad, San Diego, CA). Meanwhile, the sensitization enhancement ratio (SER) of each group was also determined by a classical multitarget single-hit model.

Cellular Uptake Study

4T1 cells were seeded on coverslips in a 24-well plate at a density of 5 × 10^4 cells per well. After culturing overnight in cell incubator (Eppendorf, CellXpert® C170, Germany), the cells were treated with Au@$AgBiS_2$-PEG with Cy5.5 as mentioned above. After incubation for 4 h, the cells were washed three times with PBS, and then the cytoskeleton and cell nuclei were stained by Alexa Fluor 488 phalloidin (green) and DAPI (blue) (Sigma-Aldrich, St. Louis, MO), respectively, according to the manufacturer's instructions. Coverslips were mounted on glass microscope slides with a drop of anti-fade mounting media (Sigma-Aldrich) to reduce fluorescence photobleaching, and then visualized by CLSM (LSM 880, Carl Zeiss Inc., Germany). For cell uptake of Ag^+ ions from Au@$AgBiS_2$-PEG, 4T1 cells were seeded in a 12-well plate at a density of 2 × 10^5 cells per well overnight. The cells were treated with Au@$AgBiS_2$-PEG with/without irradiation at different time points (0, 4h, 8h, 12h). Then the cells were collected and detected by ICP-MS to evaluate the Ag^+ ions release amount.

Immunohistochemical and Immunofluorescence Analysis in vivo

The tumor tissues were fixed in 4% formaldehyde and embedded in paraffin. Subsequently, the tumor tissues were observed *via* H&E staining. Cell proliferation and apoptosis in tumor

tissues were also analyzed by immunohistochemical staining of proliferating cell nuclear antigen Ki-67 monoclonal antibody (Santa Cruz Biotechnology, Dallas, TX) and immunofluorescence staining for TUNEL (Roche Diagnostics, Indianapolis, IN), respectively.

Immunofluorescence staining for ROS evaluation in vivo

Mice bearing 4T1 tumors were randomly divided into six groups including: (I) PBS, (II) Au, (III) Au@$AgBiS_2$, (IV) RT, (V) Au+RT, and (VI) Au@$AgBiS_2$+RT at an equivalent dose of 10 mg kg^{-1} Au and Au@$AgBiS_2$-PEG per mouse. Images of the tumor immunofluorescence slide were acquired with the automated quantitative microscopy-based image analysis system TissueFAXS Plus (TissueGnostics GmbH, Vienna, Austria) using the 20 × objective. TissueFAXS Viewer software (TissueGnostics) was used to determine the staining intensity of FITC and DAPI.

DNA damage assay

4T1 cells (5×10^4 in each well) were seeded on coverslips of 24-well plate overnight then incubated with new medium with different formulations (100 μg mL^{-1}) and further incubated at 37°C for 4 h. After replacement with fresh medium, cells were exposed to X-ray irradiation (6 Gy). After incubation for another 0, 1, 4, 12 and 24 h, the cells were then washed twice with cold PBS and fixed with 4% formaldehyde at room temperature. At 4 h post irradiation, cells were then stained with anti-γ-H_2AX mouse monoclonal antibody (dilution 1:1000) (Sigma-Aldich, USA) and anti-53BP1 mouse monoclonal antibody (Abcam Inc., MA, USA) overnight at 4°C. Rabbit anti-mouse Cy3 conjugated IgG and Rabbit anti-mouse FITC conjugated IgG (Santa Cruz Biotechnology, Dallas, TX, USA) was used as the secondary antibody to visualize the γ-H2AX and 53BP1 with 60 min incubation. CLSM was used to visualize DNA damage level by observing and counting the γ-H_2AX and 53BP1 foci.

In Vivo Distribution and ex Vivo Tumor Accumulation of Au@$AgBiS_2$-PEG

Female Balb/c mice bearing 4T1 tumor xenografts were i.v. injected with Cy5.5-labeled

$Au@AgBiS_2$. The dose of Cy5.5 was 10 mg kg^{-1} mouse body weight. At the preset times, *in vivo* fluorescence images were acquired on the Xenogen IVIS Lumina system (Caliper Life Sciences, Alameda, CA). Moreover, after 48 h postinjection, the organs of mice including heart, lung, liver, spleen, kidney, and tumor were collected. Then fluorescence images were also acquired on the Xenogen IVIS Lumina system as well.

In vitro cell viability assay

CCK-8 assays were used to evaluate the cytotoxicity of $Au@AgBiS_2$. 4T1 cells were seeded on 96-well plates with a density of 5×10^3 cells per well. After incubating for 24 hours, various concentrations of were $Au@AgBiS_2$-PEG added into 96-well plates for 4 h incubation. The cells were irradiated by 6 Gy X-ray or not. After incubation for 24 hours, 10 μL 5mg mL^{-1} CCK-8 solution were added for another 4 h incubation. The medium was discarded and 100 μL The absorbance was measured at 488 nm on a microplate reader.

Animals and Tumor Model

Female Balb/c mice at 6-8 weeks of age were obtained from Vital River Laboratories (Beijing, China). All animals received care in compliance with the guidelines outlined in the Guide for the Care and Use of Laboratory Animals, and all procedures were approved by Anhui Medical University Animal Care and Use Committee. The xenograft tumor model was generated by injection of 1×10^6 4T1 cells (100 μL) into the right flank of Balb/c mice.

Tumor Suppression Study

For RT treatment using with various concentrations of Au and $Au@AgBiS_2$, when the tumor volumes were around 100 mm^3, the 4T1 tumor-bearing mice were i.v. injected with various formulations (n = 5). At 12 h post-injection, the mice were received X-ray irradiation at dose of 6 or 8 Gy. The tumor growth was monitored by measuring the perpendicular diameter of the tumors (i.e., length and width, respectively) using calipers every 2 days. The estimated volume was calculated according to the formula: tumor volume (mm^3) = $0.5 \times$ length $\times$ $width^2$. Weight of each mouse was also measured every 2 days.

Flow-cytometry cell apoptosis detection

4T1 cells were seeded into 6-well plates at the density of 2×10^5 cells per well (1 mL of RPMI 1640 medium) and then incubated with DMSO, Au NPs in DMSO (the final concentrations in RPMI 1640 medium is 100 μg mL^{-1}) and Au@$AgBiS_2$-PEG in DMSO (the final concentrations in RPMI 1640 medium is 100 μg mL^{-1}) for 24 h, respectively. The single cell suspension was further obtained and stained with annexin-FITC and prodium iodide (PI) for flow cytometry (CytoFLEX LX, Beckman Counter).

Cell morphology observation

4T1 cells were seeded into 6-well plates at the density of 2×10^5 cells per well (1 mL of RPMI 1640 medium) and then incubated with DMSO, Au NPs in DMSO (the final concentrations in RPMI 1640 medium is 100 μg mL^{-1}) and Au@$AgBiS_2$-PEG in DMSO (the final concentrations in RPMI 1640 medium is 100 μg mL^{-1}) for 24 h, respectively. The cells were irradiated by irradiation 6 Gy or not. The morphology of cells was further observed by inverted fluorescence microscope (Olympus, Japan). Real-time 4T1 cells morphology images treated with Au@$AgBiS_2$-PEG combined RT were observed for 48 h *via* using full-automatic live cell imaging system (Cell discoverer 7.0).

Enzyme linked immunosorbent assay (ELISA) for detection of the cytokines in serum

Tumors excised from Balb/c mice were pulverized and treated with PBS buffer for 10 min at 4°C. The mixture was centrifuged at 5000 g at 4°C for 10 min, and the supernatants were collected for ELISA detection according to the manufacturer's instructions.

Irradiation

The mice were irradiated with 6 MV X-rays generated by a multifunctional electron linear accelerator Varian 23EX, which was used for clinical radiotherapy at the Department of Radiation Oncology, the First Affiliated Hospital of Anhui Medical University. The dose rate was 6 Gy min^{-1}, and the distance from the source to the skin (SSD) is 100 cm. The irradiation site is the entire dorsal skin of the mouse. Before the experiments, we used a model to calculate

the energy distribution of the X-ray, and found that the maximum energy depth is 1.6 cm. The mouse dorsal tumor was covered with 1 cm solid water film (bolus) to make sure that most X-ray energy were deposited at the skin.

Lactate dehydrogenase (LDH) release assay

The LDH released was assayed with an LDH Cytotoxicity Assay Kit (C0017, Beyotime, Shanghai, China). In brief, the cells were irradiated and cultured for 48 h, and the culture medium was then replaced with fresh culture medium with 1% FBS. At 72 h after irradiation, the supernatant of the cell culture was collected for the detection of LDH activity according to the manufacturer's protocol.

Western blotting for detection of cleaved caspase3 , GSDME and NLRP3 related proteins.

The cells were collected by centrifugation and lysed in RIPA buffer with PMSF (1 mM). The protein concentration was determined with a BCA Protein Assay Reagent Kit (Beyotime Biotechnology, Shanghai, China). The cell protein extracts were subjected to SDS–PAGE, transferred onto polyvinyl difluoride membranes, blocked with 5% nonfat dry milk and subsequently incubated with primary antibodies at 4°C overnight. The membranes were incubated with secondary antibodies for 1 h, and signals were detected with Tanon® 1600 Infrared Imaging System (Tanon, Shanghai, China).

Detection of Calreticulin

4T1 cells 5×10^4 were seeded in a 24-well plate overnight , and then treated with different formulations for 4 h, following 6 Gy irradiation or not. Following a further incubation of 4 h, CRT exposure on the surface of 4T1 cells after different treatments were detected by flow cytometry using Rabbit anti-Calreticulin antibody to CRT (1:300, ab18847, Abcam, USA).

Detection of HMGB1

4T1 cells 5×10^4 were seeded in a 24-well plate overnight and then treated with different formulations for 4 h, following 6 Gy irradiation or not. Following a further incubation of 12 h, cells were incubated with individual primary antibodies against HMGB1 antibody (1:500,

ab18256, Abcam, USA) for overnight at 4°C, and followed washed three times with PBS, and then the HMGB1 and cell nuclei were stained by Alexa Fluor 594-labeled goat anti-mouse IgG (Abcam, USA) and DAPI, according to the manufacturer's instructions. Coverslips were mounted on glass microscope slides with a drop of anti-fade mounting media, and then visualized by CLSM.

Detection of the concentration of extracellular ATP

4T1 cells 5 × 10^4 were seeded in a 24-well plate overnight and then treated with different formulations for 4 h, following 6 Gy irradiation or not. After a further incubation of 12 h, extracellular ATP levels in the DMEM media were detected *via* ATP bioluminescent assay kit (Beyotime Biotechnology) by Xenogen. IVIS Spectrum imaging system (Perkin Elmer, Waltham, MA, USA).

Cell Surface Markers by FACS detection

The spleen pieces obtained for single-cell analysis were gently meshed though nylon mesh. Red blood cells were lysed by Ack lysing buffer (Biosharp) according to the manufacturer's instructions. Cell preparations were stained for 30 min at 4ºC with cocktails containing combinations of fluorochrome-conjugated monoclonal antibodies for cell surface markers. Then extracellular staining with antibodies (containing FITC anti-mouse CD3ε, APC anti-mouse CD4, APC/Cyanine7 anti-mouse CD8a, Brilliant Violet 605™ anti-mouse/human CD44, PE anti-mouse CD62L, PE/Cyanine7 anti-mouse CD11c, FITC anti-mouse CD80, APC anti-mouse CD86; all antibodies were purchased from BioLegend) were added into cell samples. The obtained cells and antibodies solution was stored in 4ºC for 1 h in the dark. After that, the cells were washed with PBS twice and then dispersed in 0.5 mL of PBS for surface marker detection directly by flow cytometry (CytoFLEX LX, Beckman Counter).

Preparation of BMDCs. Bone marrows of femurs and tibias were flushed and erythrocytes were lysed. The obtained cells were suspended at a concentration of 1 × 10^6 cells/mL in BMDC growth media (RPMI 1640 containing 10% FBS, penicillin-streptomycin, sodium pyruvate, 20

ng/mL GM-CSF, and 10 ng/mL IL-4). Culture medium of BMDCs was replaced in half every other day. On day 6, non-adherent cells and loosely adherent cells were collected and the purity of DCs was determined by flow cytometric analysis of CD11c expression.

Lung Metastases Prevention Study

When the tumor volumes were around 100 mm^3, the 4T1 tumor-bearing mice were i.v. injected with congeneric Luc-4T1 tumor cell line that stably express luciferase (5×10^4 per mouse). The 4T1 tumor-bearing mice were then i.v. injected with various formulations (n = 6 for each group) at 12 h after injection. The mice were received irradiated with a singer fraction of 6 Gy. Mice were i.p. injected with substrate (D-luciferin potassium salt, 150 mg kg^{-1} body weight) before bioluminescence imaging using an in vivo imaging instruments (IVIS) spectrum system. Besides, lungs were analyzed ex *vivo* and hematein and eosin staining at the end of therapy.

References

[1] W. N. Wang, P. Pei, Z. Y. Chu, B. J. Chen, H. S. Qian, Z. B. Zha, W. Zhou, T. Liu, M. Shao, H. Wang, *Chem. Eng. J.* **2020**, *397*, 125488.

[2] C. Wang, L. Cheng, Z. Liu, *Biomaterials* **2011**, *32,* 1110.

[3] W. Jiang, Q. Li, L. Xiao, J. X. Dou, Y. Liu, W. H. Yu, Y. C. Ma, X. Q. Li, Y. Z. You, Z. T. Tong, H. Liu, H. Liang, L. G. Lu, X. D. Xu, Y. D. Yao, G. Q. Zhang, Y. C. Wang, *ACS Nano* **2018**, *12,* 5684.

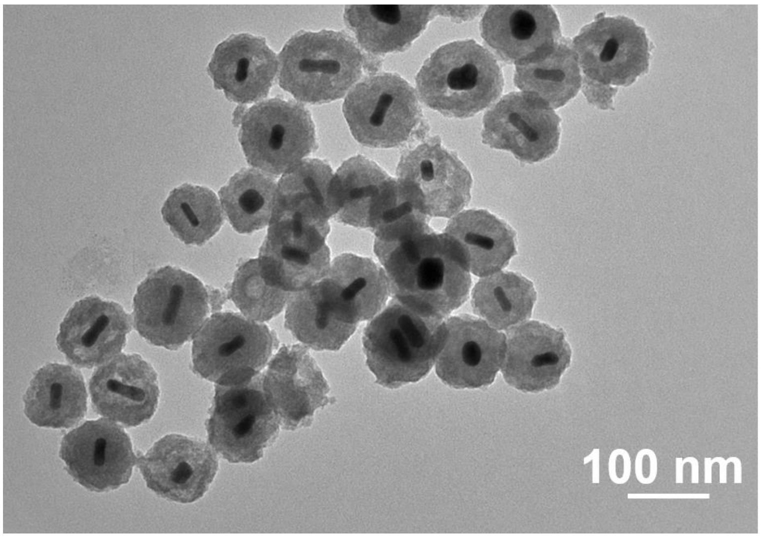

Figure S1. TEM images of the Au@ZnS core-shell structure nanoparticles, scale bars = 100 nm.

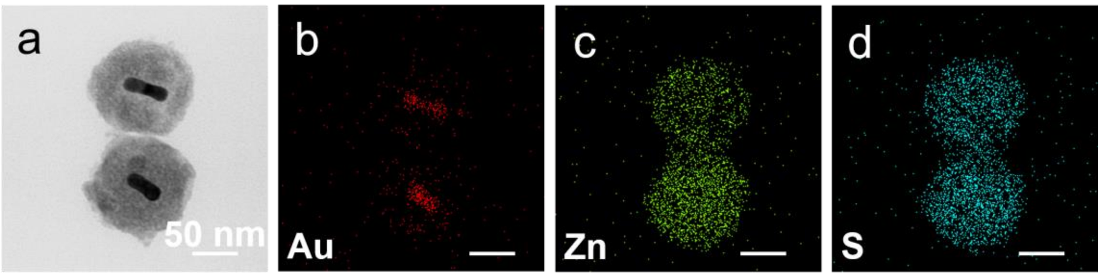

Figure S2. (a-d) Scanning transmission electron microscopy (STEM) images of Au@ZnS core-shell structure nanoparticles and the elements mappings of Au, Zn, and S, scale bars = 50 nm.

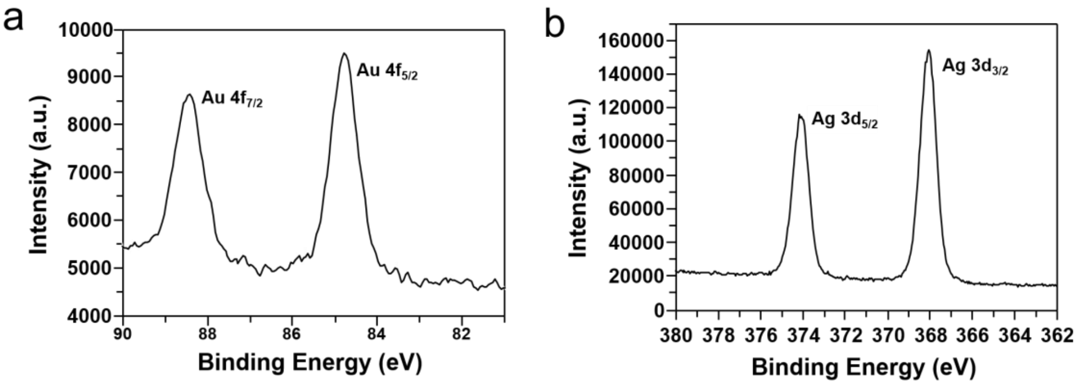

Figure S3. X-ray photoelectron spectra of the Au 4f (a) and Ag 3d (b) peaks.

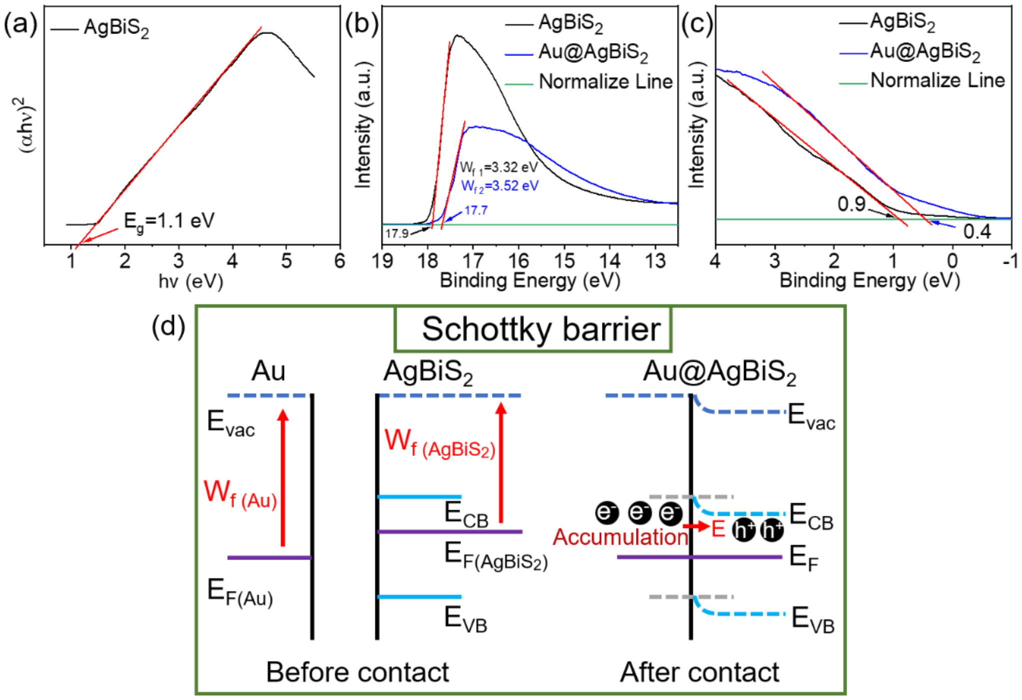

Figure S4. (a) Ultraviolet-visible absorption spectra with the Tauc analyses. (b) Photoemission cutoff spectra and (c) Valence band (VB) structure of $AgBiS_2$ and $Au@AgBiS_2$. E_g, band gap; W_f, work function. (d) Schottky barrier diagram. E_{vac}, vacuum level; E_F, fermi level; E_{CB}, conduction band; E_{VB}, valence band.

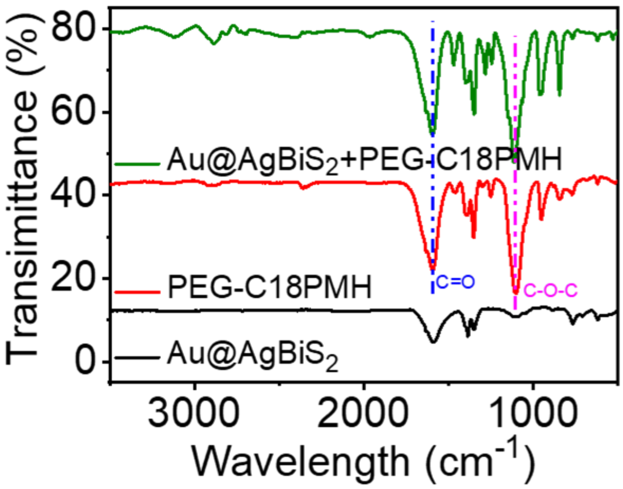

Figure S5. FT-IR spectra of $Au@AgBiS_2$, PEG-C_{18}PMH, and $Au@AgBiS_2$-PEG.

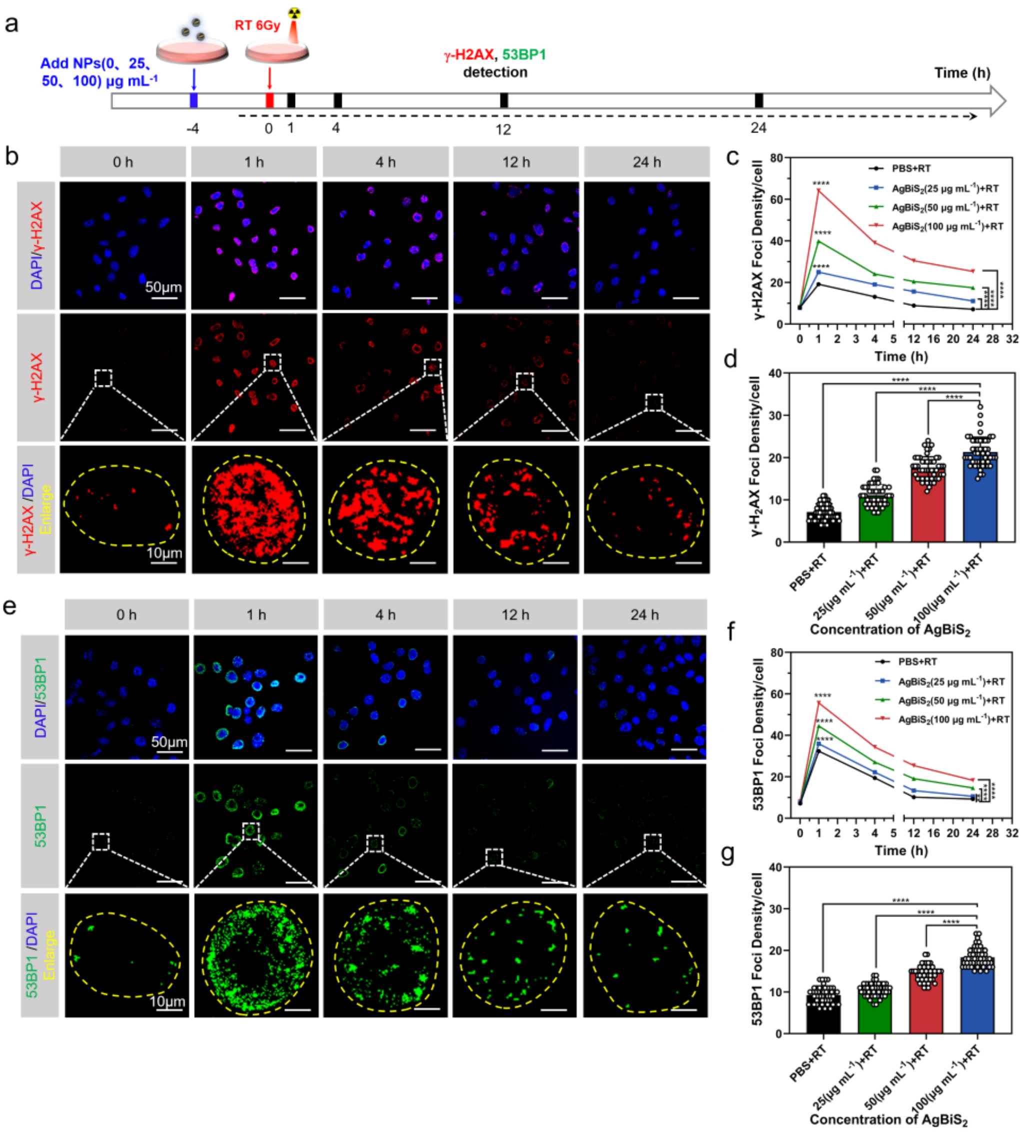

Figure S6. $AgBiS_2$ potentiated RT-induced DNA damage in a dose-dependent manner. The cells were cultured with various doses of $AgBiS_2$ for 4 hours, followed by X-ray irradiation (6 Gy). Quantitative analysis of γ-H_2AX foci and 53BP1 foci in 4T1 tumor cellular nuclei at various time points (0 h, 1 h, 4 h, 12 h, and 24 h) post RT treatment (in terms of number of γ-H_2AX foci per cell, n = 50 cells). (a) Treatment plan for RT with 4T1 cells pretreated with $AgBiS_2$. (b,e) The confocal images of γ-H_2AX foci (red), 53BP1 foci (green) and cell nuclei (blue) by immunofluorescence staining. (c,f) The quantitative density of γ-H_2AX foci and 53BP1 foci in 4T1 cells preincubated with different doses of $AgBiS_2$ at various time points. (d,g) Quantitative calculation of γ-H_2AX foci and 53BP1 foci in 4T1 tumor cellular nuclei at 24 h post RT treatment. The experiment was repeated three times independently. (*$P < 0.05$,

**P < 0.01, ***P < 0.001, ****P < 0.0001).

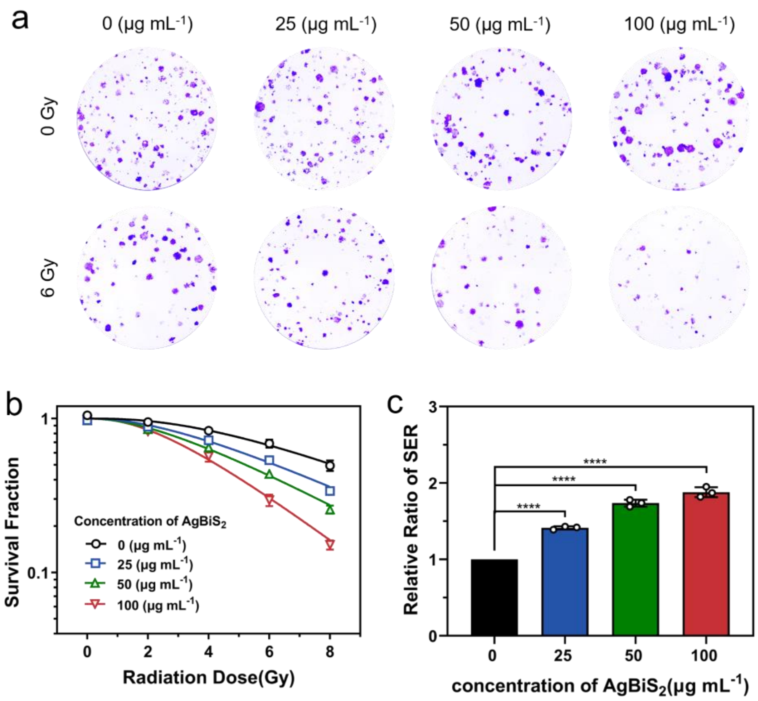

Figure S7. $AgBiS_2$ enhanced the radiosensitivty to 4T1 cells in vitro. The cells were cultured with various doses of $AgBiS_2$ for 4 hours, followed by a single dose of X-ray irradiation (6 Gy). (a) Representative images of colony formation of 4T1 cells treated with $AgBiS_2$ under X-ray radiation (6 Gy) condition. (b) Cell survival curves of 4T1 cells pretreated the different concentration of $AgBiS_2$ under various doses of RT treatment condition. (c) The SER values of each group were determined by single-hit multitarget mode (n = 3). The experiment was repeated three times independently. (*P < 0.05, **P < 0.01, ***P < 0.001, ****P < 0.0001).

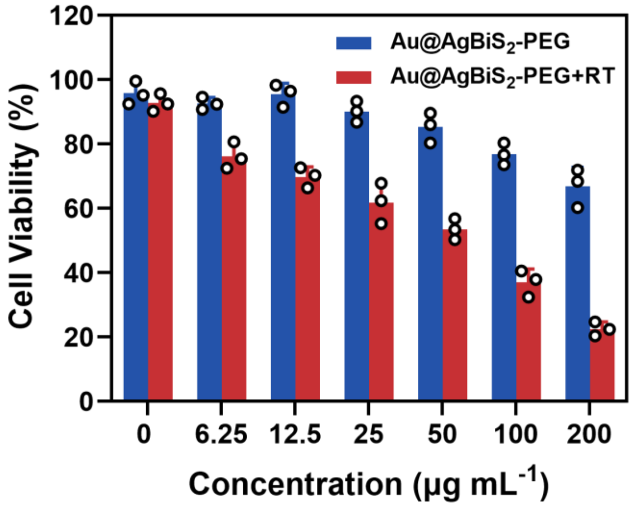

Figure S8. Evaluating the cytotoxicity of Au@AgBiS$_2$-PEG with irradiation 6 Gy or not by CCK-8 assay. Data are shown as mean ± SD (n = 3). The experiment was repeated three times independently. (*P < 0.05, **P < 0.01, ***P < 0.001, ****P < 0.0001).

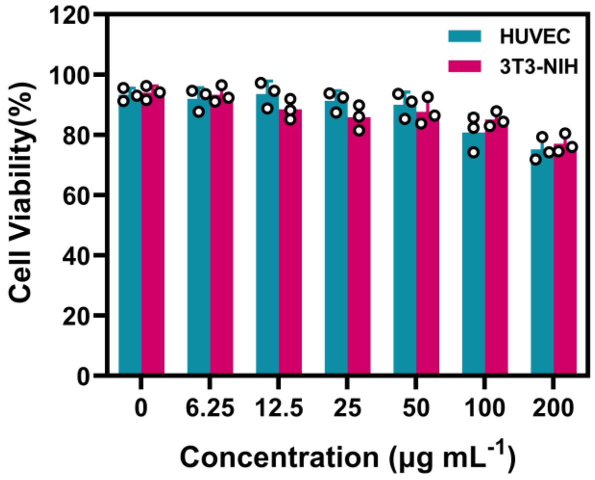

Figure S9. Relative viability of HUVEC and 3T3-NIH cells treated with Au@AgBiS$_2$-PEG and with fresh medium for additional 12 h. The cell viabilities were measured by CCK-8 assay. Data are shown as mean ± SD (n = 3). The experiment was repeated three times independently. (*P < 0.05, **P < 0.01, ***P < 0.001, ****P < 0.0001).

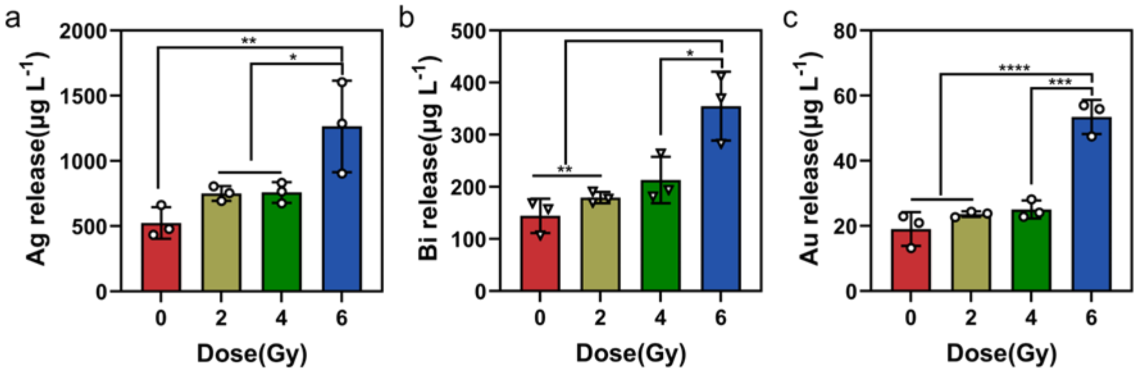

Figure S10. Ag ion, Bi ion ang Au ion of Au@$AgBiS_2$-PEG subject to various irradiation doses (0 Gy, 2 Gy, 4 Gy and 6 Gy) by ICP-MS for quantitative analysis (n = 3). (*P < 0.05, **P < 0.01, ***P < 0.001, ****P < 0.0001).

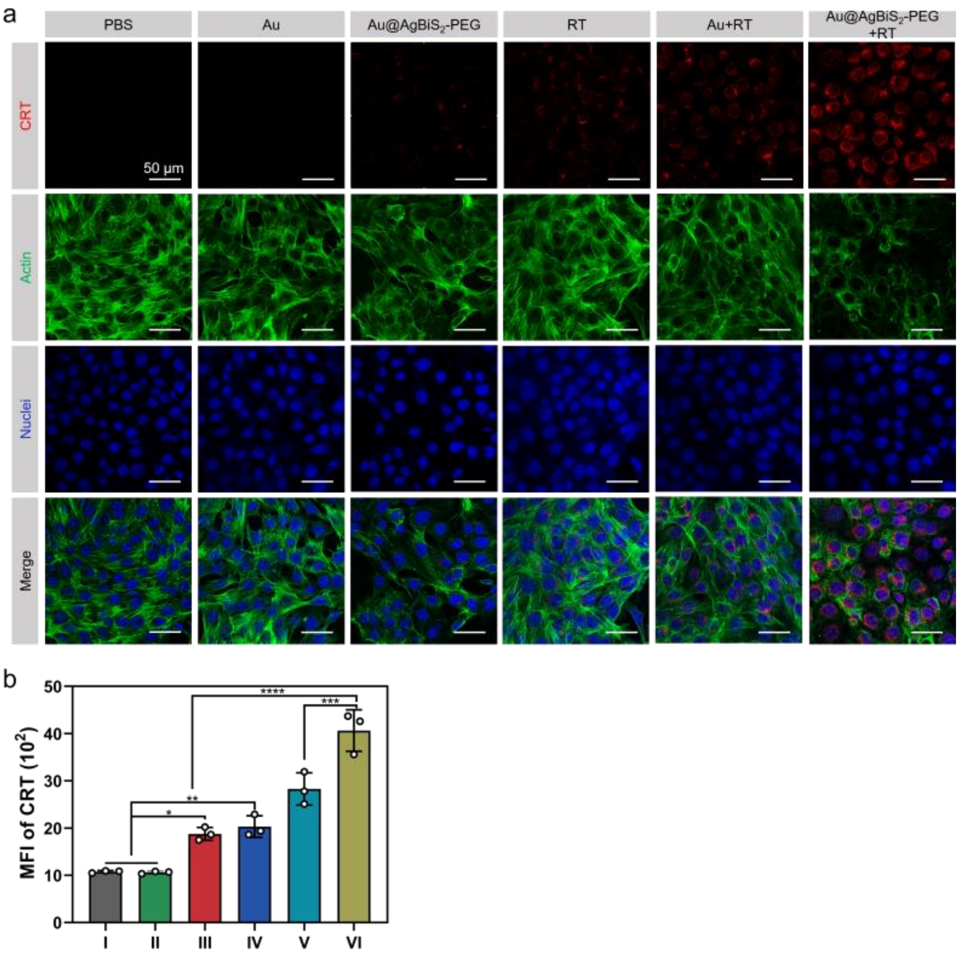

Figure S11. (a) Confocal microscopy showing the induction of the CRT in 4T1 tumors at 4 h after irradiation 6 Gy. The cytoskeleton (Actin) and cell nuclei were stained by Alexa Fluor 488 phalloidin (green) and DAPI (blue), respectively (scale = 50 μm) (n = 3). (b) The mean fluorescence intensity (MFI) quantification of CRT in 4T1 tumor cells. (*P < 0.05, **P < 0.01, ***P < 0.001, ****P < 0.0001).

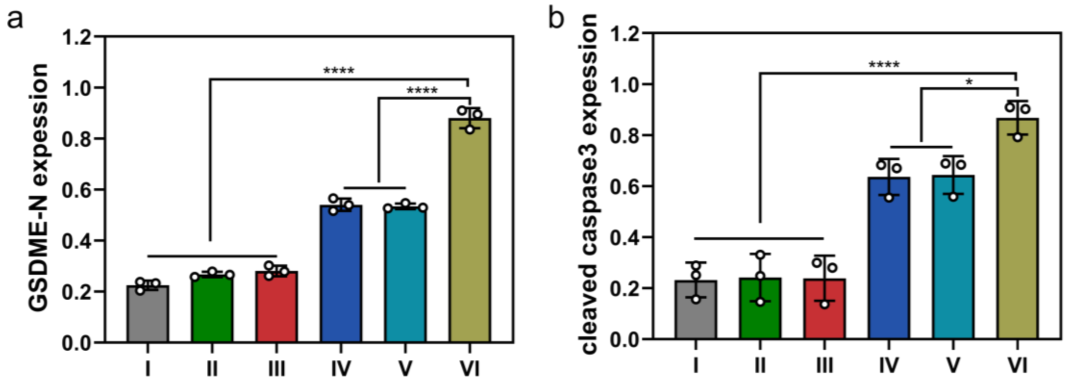

Figure S12. Quantitative analysis for relative expression levels of GSDME-N (a) and cleaved caspase3 (b) by western blot (n=3). The experiment was repeated three times independently. (*$P < 0.05$, **$P < 0.01$, ***$P < 0.001$, ****$P < 0.0001$).

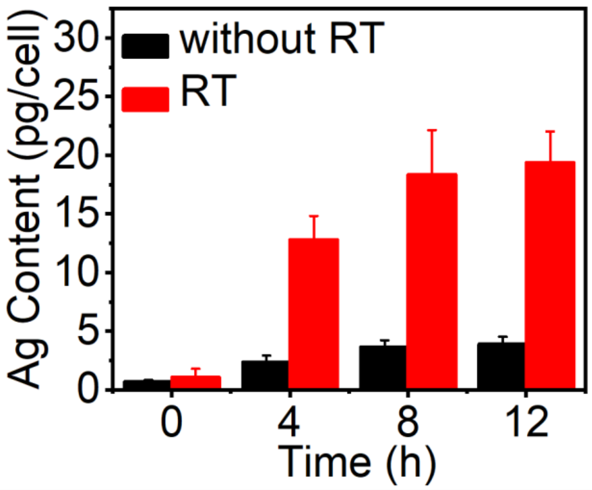

Figure S13. Cell uptake of Ag^{+} ions from Au@$AgBiS_2$-PEG at different time points under RT and non-RT treatment. (n=3)

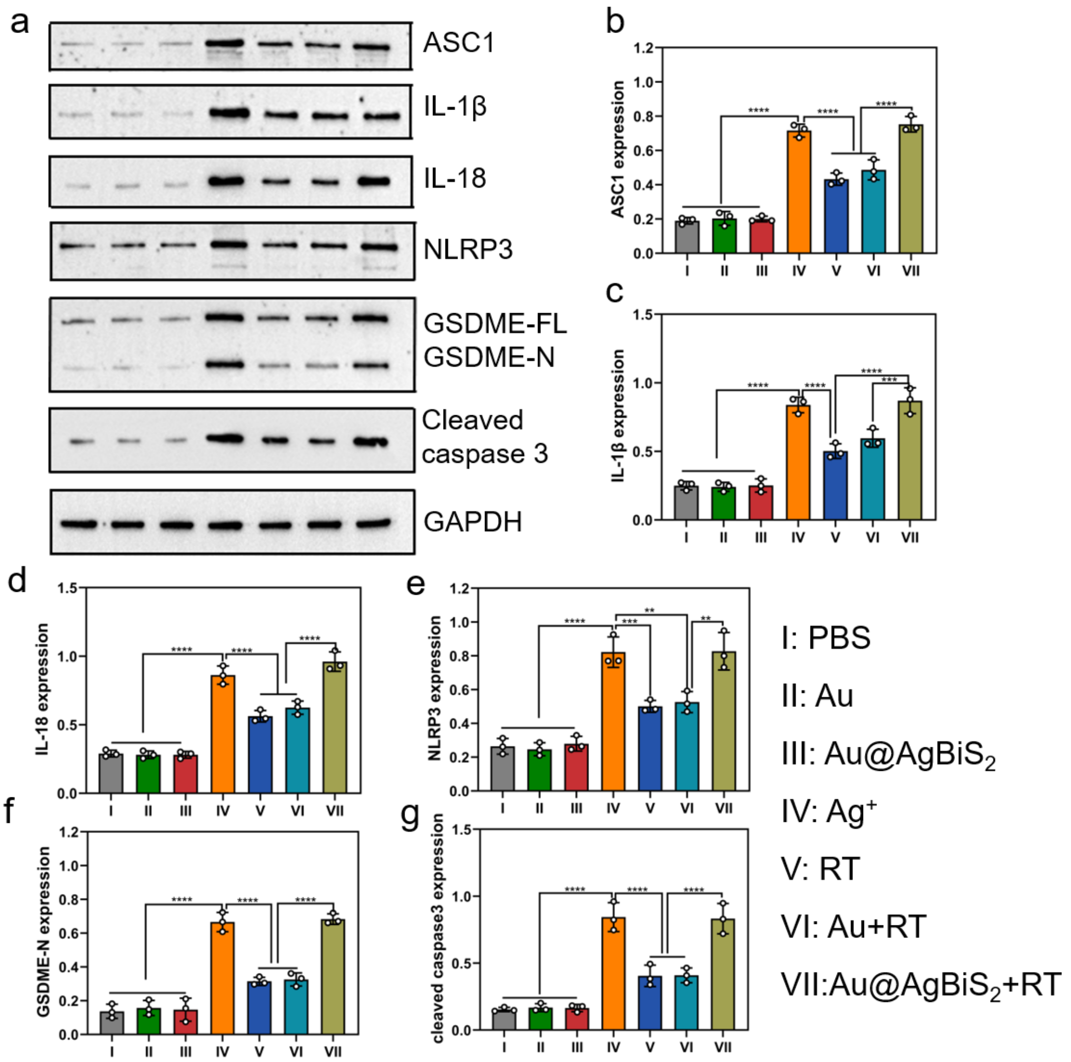

Figure S14. The Au@AgBiS$_2$-PEG under irradiation upregulated the levels of NLRP3 indicators (ASC1, IL-1β, IL-18, and NLRP3) and pyroptotic protein. (a) The expression of NLRP3 indicators and pyroptotic protein by western blot. (b-g) Quantitative analysis of NLRP3 related genes and pyroptotic protein. The experiment was repeated three times independently. Data are shown as mean ± SD by one-way analysis of variance (ANOVA) was used. (*P < 0.05, **P < 0.01, ***P < 0.001, ****P < 0.0001).

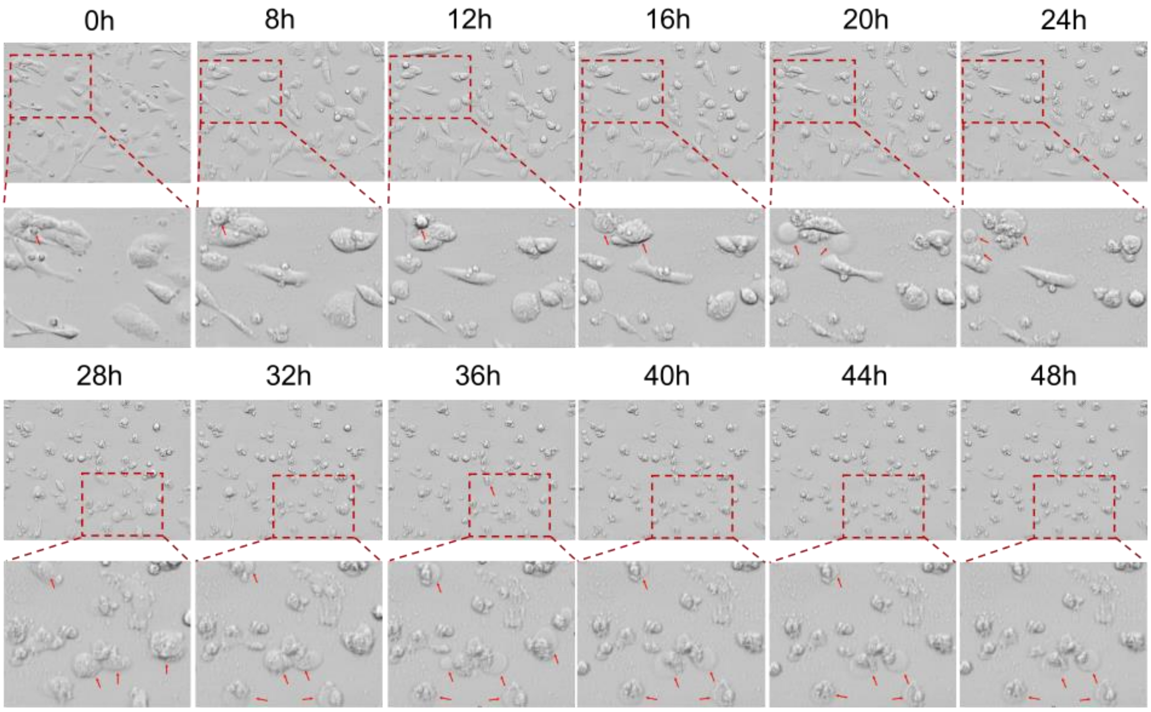

Figure S15. 4T1 cells morphology images treated with Au@AgBiS_2-PEG combined with RT for different time intervals. Cell swelling with large bubbles can be observed in Au@AgBiS_2+RT (Group VI) by the full-automatic live cell imaging system (Cell discoverer 7.0). Red arrows of magnification images indicate balloon-like pyroptotic cells at various time points. (n=3).

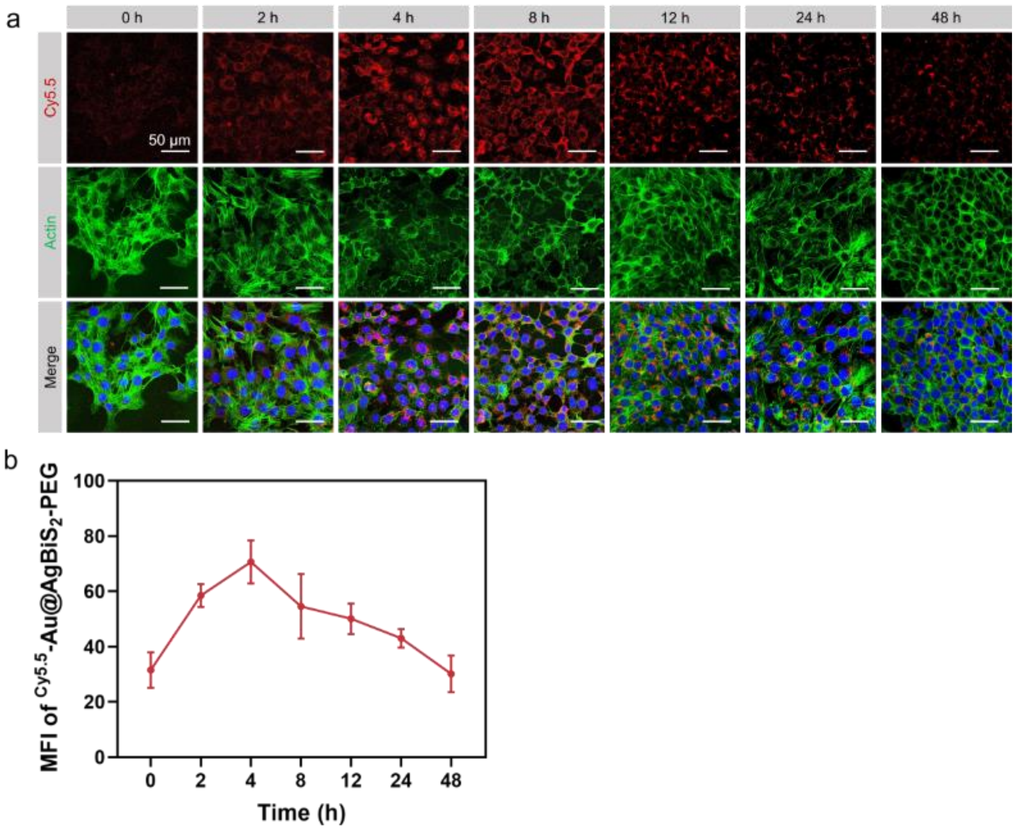

Figure S16. (a) CLSM observation of 4T1 cells incubated with Cy5.5-labeled Au@$AgBiS_2$-PEG for different time points (0 h, 2 h, 4 h, 8 h, 12 h, 24 h and 48 h). (b) MFI of Cy5.5-labeled Au@$AgBiS_2$-PEG by Quantitative analysis Mean Fluorescence Intensity (MFI). The cytoskeleton (Actin) and cell nuclei were stained by Alexa Fluor 488 phalloidin (green) and DAPI (blue), respectively (scale = 50 μm) (n = 3). (*$P < 0.05$, **$P < 0.01$, ***$P < 0.001$, ****$P < 0.0001$).

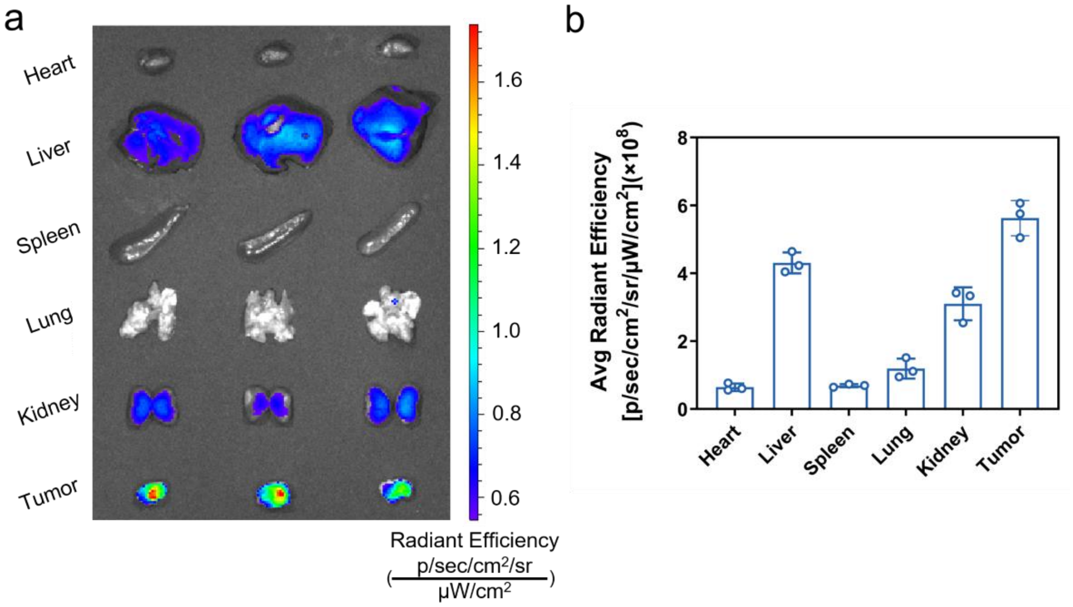

Figure S17. (a) Fluorescence imaging of major organs (heart, liver, spleen, lung, and kidney) and tumor of the 4T1 tumor bearing mice. Images were taken at 48 h post-injection of Au@AgBiS_2-Cy5.5; (b) Mean fluorescence intensity analysis of the major organs and tumor obtained from panel a (n = 3). (*P < 0.05, **P < 0.01, ***P < 0.001, ****P < 0.0001).

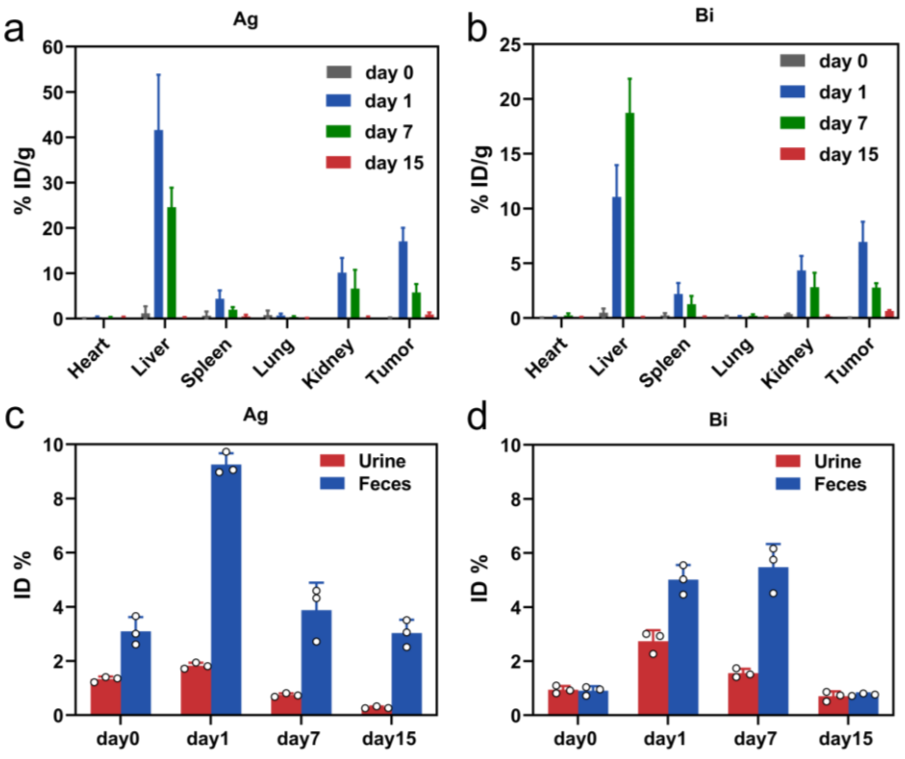

Figure S18. Biodistribution of Au@AgBiS$_2$-PEG post intravenous injection. (a, b) The time dependent Ag ion and Bi ion of Au@AgBiS$_2$-PEG distribution in organs (including the tumor) and (c, d) metabolites (urine and feces) by ICP-MS-detected Ag iron and Bi iron concentrations in mice bearing 4T1 tumors at different time points (day 0, day 1, day 7 and day 15) (n = 3).

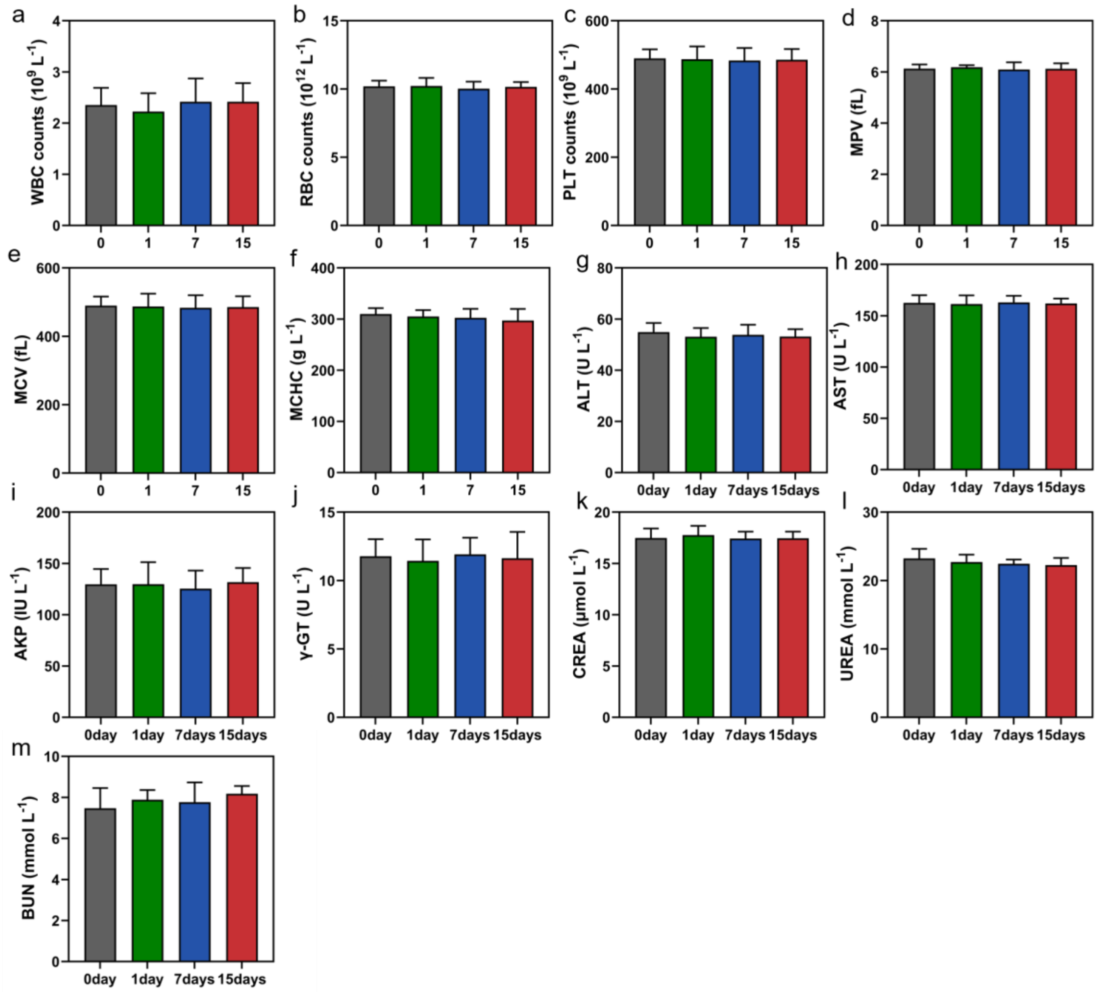

Figure S19. Quantitative analysis of routine blood (a-f) and blood biochemical (g-k) of the 4T1 tumor-bearing mice by i.v. injected with Au@AgBiS_2-PEG for different time points (day 0, day 1, day 7 and day 15). WBC: white blood cell; RBC: red blood cell; PLT: platelets; MPV: mean platelet volume; MCV: mean corpuscular volume, MCHC: mean corpuscular hemoglobin concentration; ALT: Alanine aminotransferase; AST: Aspartate aminotransferase; AKP: alkaline phosphatase; γ-GT: Gamma-glutamyltransferase; PCT: Platelet cubic measure distributing width; UREA: carbamide; CREA: Creatinine; BUN: blood urea nitrogen. (n = 5).

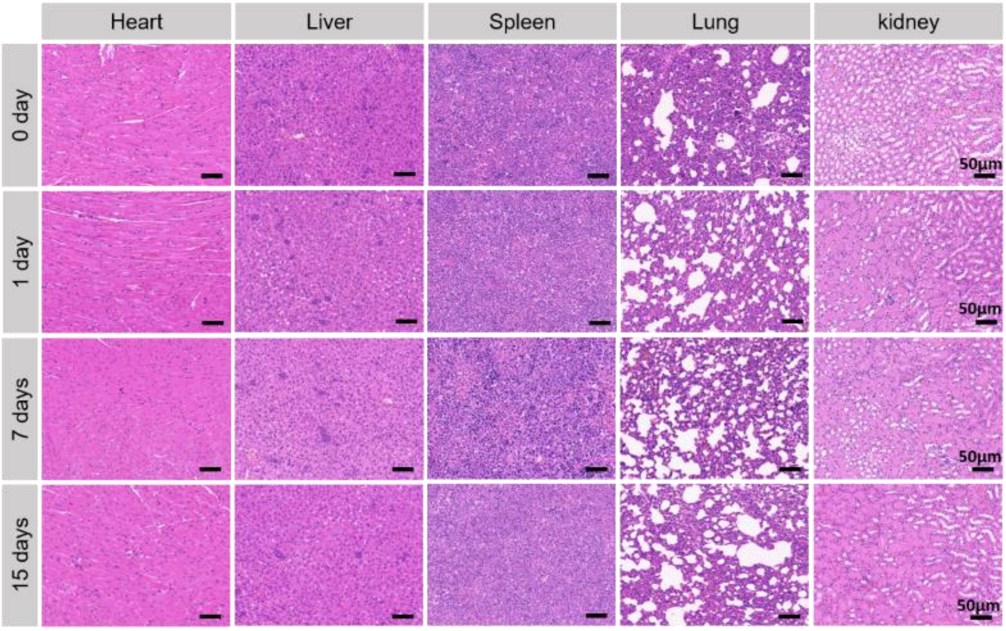

Figure S20. Observation of major organs (heart, liver, spleen, lung, and kidney) by H&E staining post i.v injected with Au@$AgBiS_2$-PEG at various time points (day 0, day 1, day 7 and day 15) (n = 5) (scale bar = 50 μm).

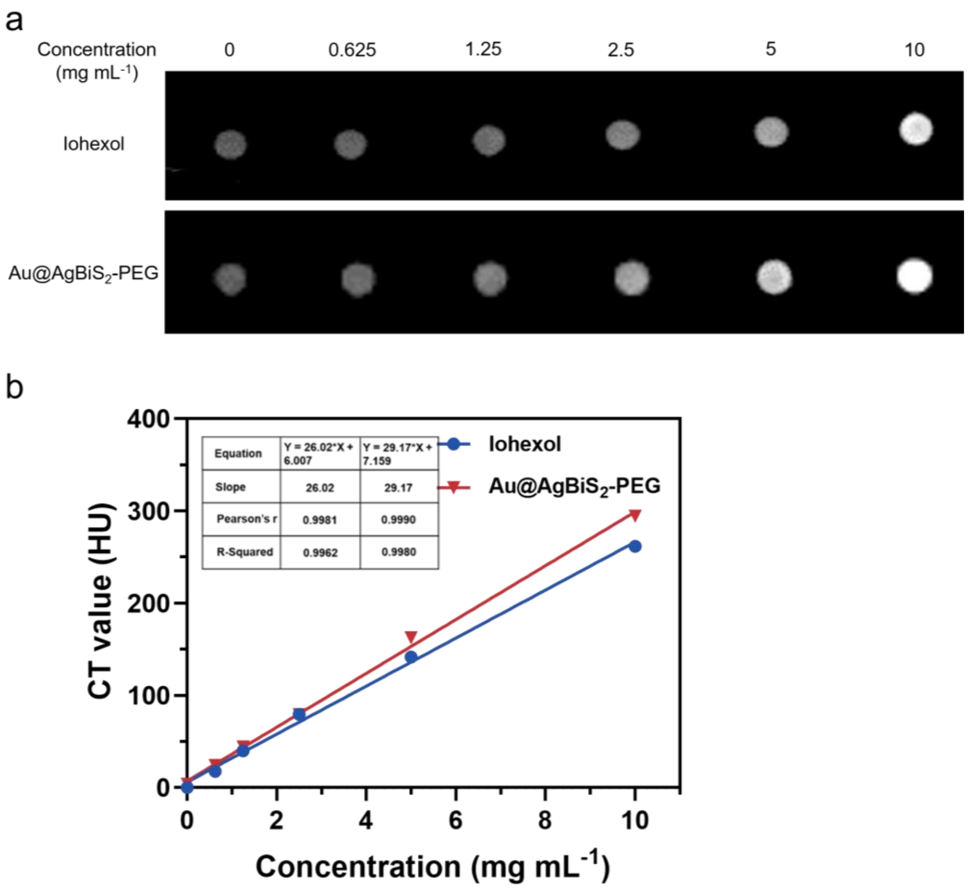

Figure S21. (a) In vitro CT images of the Au@AgBiS$_2$-PEG with different concentrations compared with Ioxehol; (b) CT values of as prepared Au@AgBiS$_2$-PEG in accordance with the concentrations (n = 3). Data are shown as the mean ± SEM (n = 3). *P < 0.05, **P < 0.01, ***P < 0.001, ****P < 0.0001.

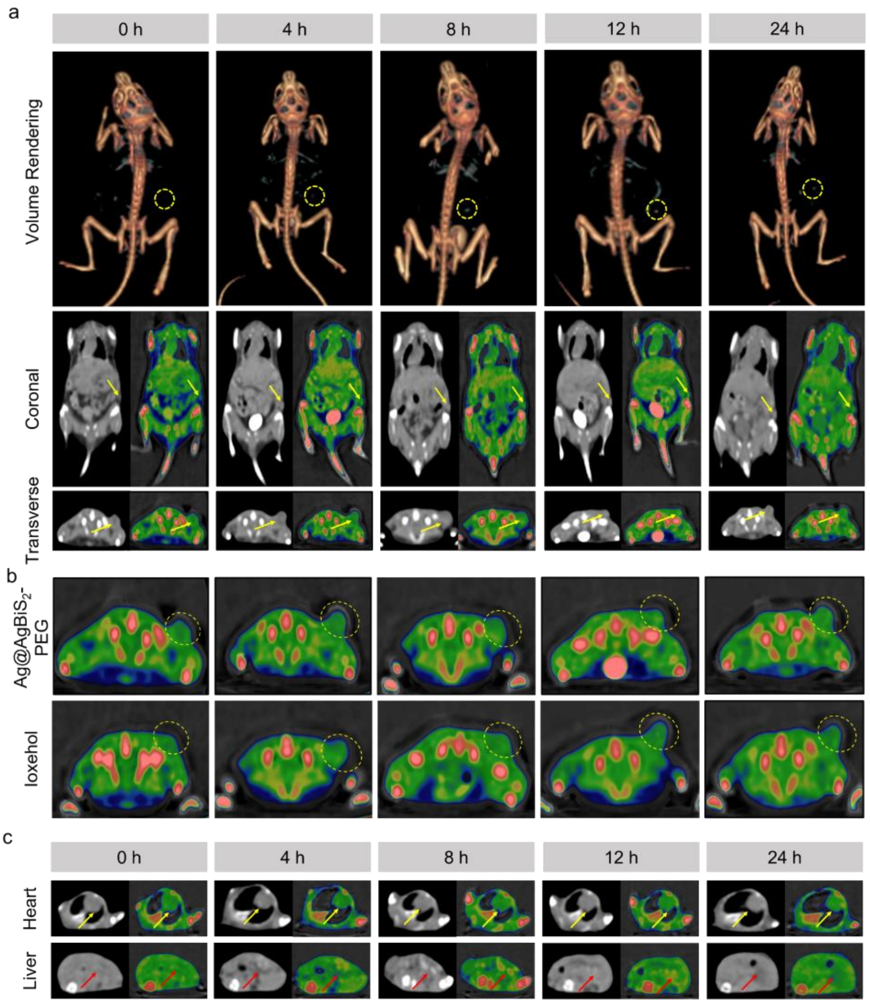

Figure S22. (a) In vivo volume rendering (VR) images, coronal and transverse sections of tumor area of 4T1-tumor-bearing mice after intravenous injection of the Au@$AgBiS_2$-PEG solution at different time points (0 h, 4 h, 8 h, 12 h, and 24 h). Yellow dashed circles (top) and yellow arrows (middle, bottom) indicate the tumor regions (n = 3). (b) The contrast enhancement CT images (magnification images from S15a) of 4T1 tumor-bearing mice following intravenous injection Au@$AgBiS_2$-PEG and Iohexol at the above time points. Yellow dashed circles indicate the tumor areas. (c)The CT images of heart and liver with 4T1 tumor-bearing mice post intravenous injection of the Au@$AgBiS_2$-PEG solution at the various time intervals. Yellow arrows and red arrows show the heart and liver, respectively. The grayscale images and MIP fusion color images are showed at left column and right column, respectively.

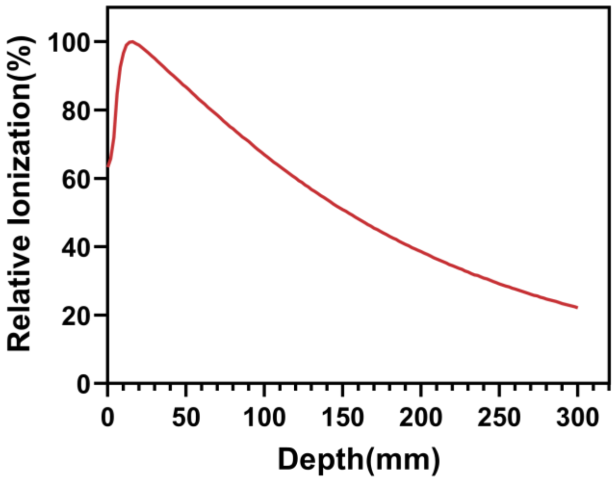

Figure S23. Percentage depth dose (PDD) measurement in water by IBA three-dimensional dose monitoring system(scanning profile : 40 cm×40 cm ×40 cm).

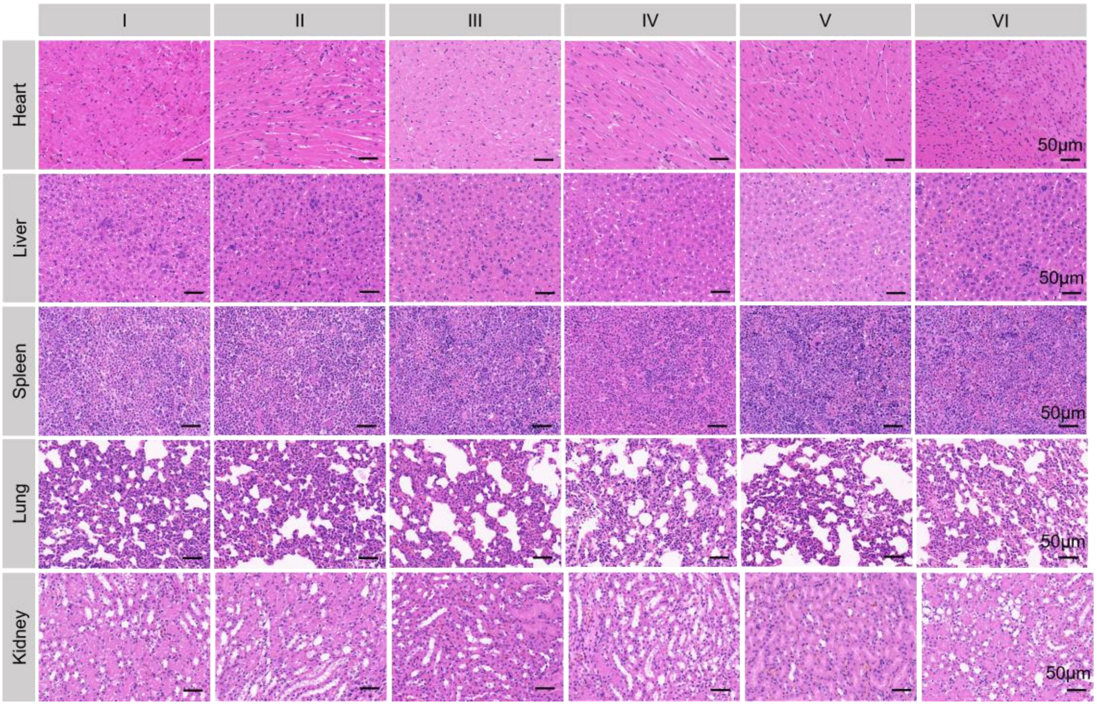

Figure S24. The major organs (heart, liver, spleen, lung, and kidney) were collected at the end of Antitumor assay and observed by H&E staining to evaluation of different treatment (n = 3).

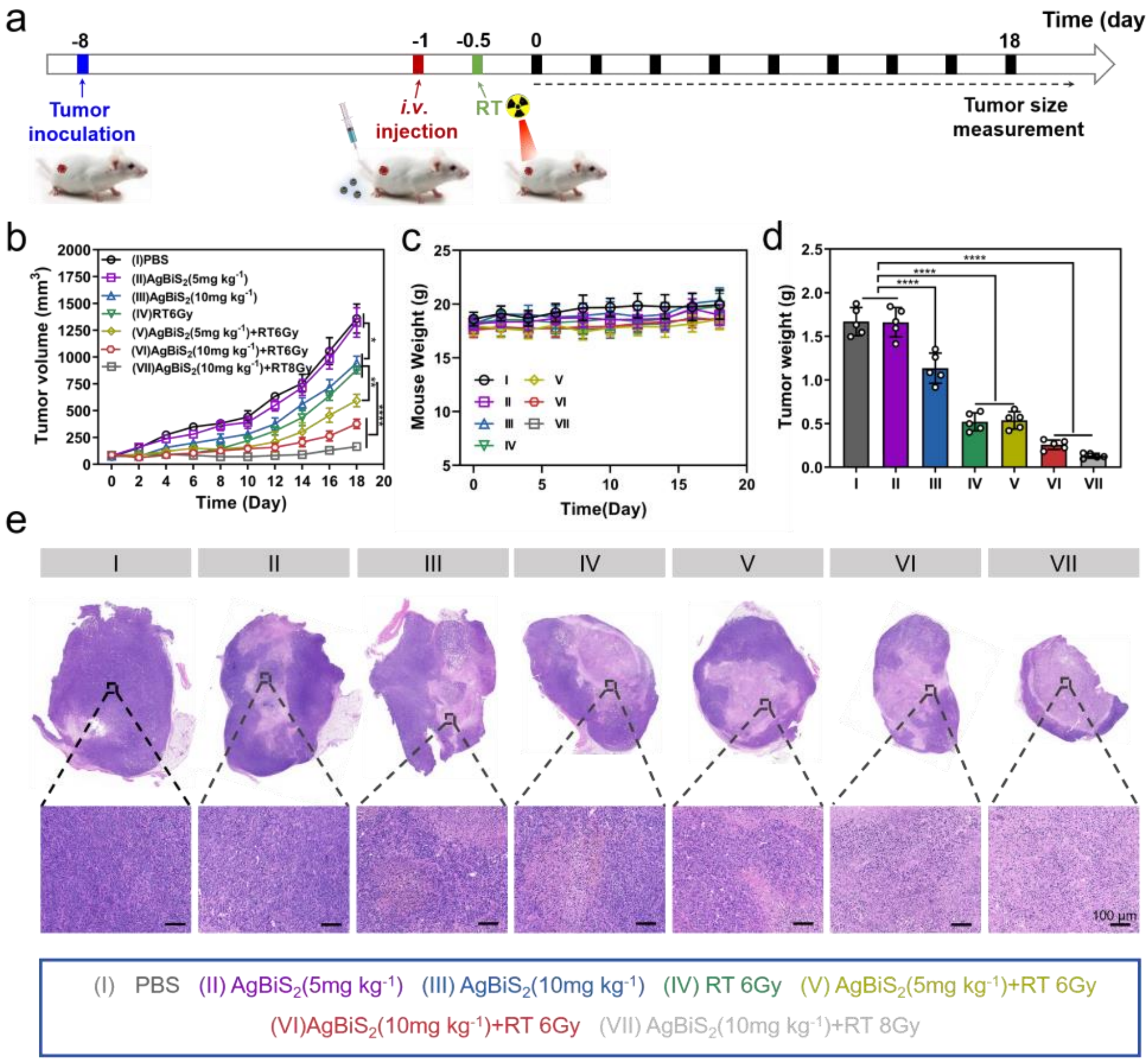

Figure S25. $AgBiS_2$ enhanced the radiosensitivty to 4T1 cells *in vivo.* (a) Treatment plan for the $AgBiS_2$ improved RT-mediated antitumor efficacy of 4T1 tumor-bearing mice. (b) The changes of tumor volume for various groups after irradiation as shown. (c) The analysis of tumor weights on the day 18 in every groups. (d) The monitoring of body weight fluctuation of mice during treatment process. (e) H&E staining of the whole tumor after treatments. Scale bars = 100 μm. (n = 5). All the data were expressed as mean ± SEM. (*P < 0.05, **P < 0.01, ***P < 0.001, ****P < 0.0001).

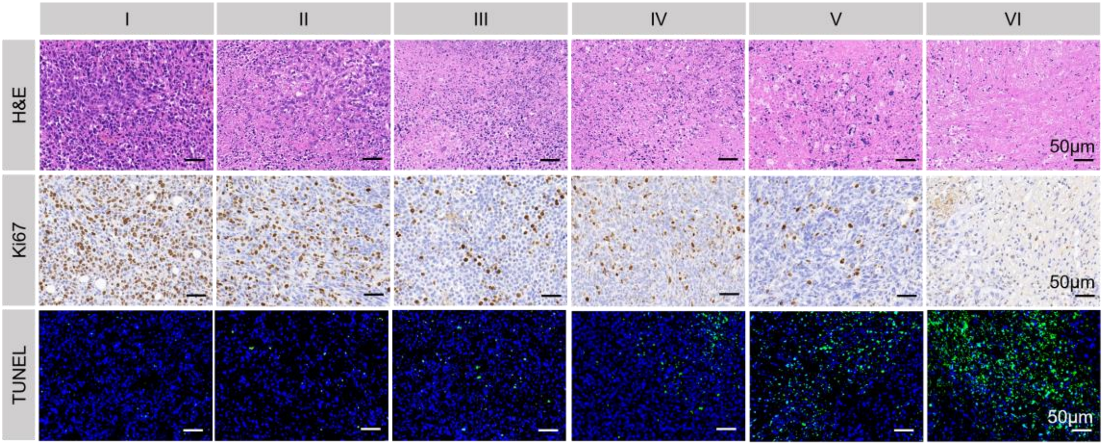

Figure S26. Representative H&E, TUNEL, and Ki67 staining images of tumor tissue received the different treatments. TUNEL positive apoptosis cells were stained green, and cell nuclei were constrained blue. Ki67-positive proliferating cells were stained brown and apoptotic cells were stained blue, respectively. (scale = 50 μm). (n = 5).

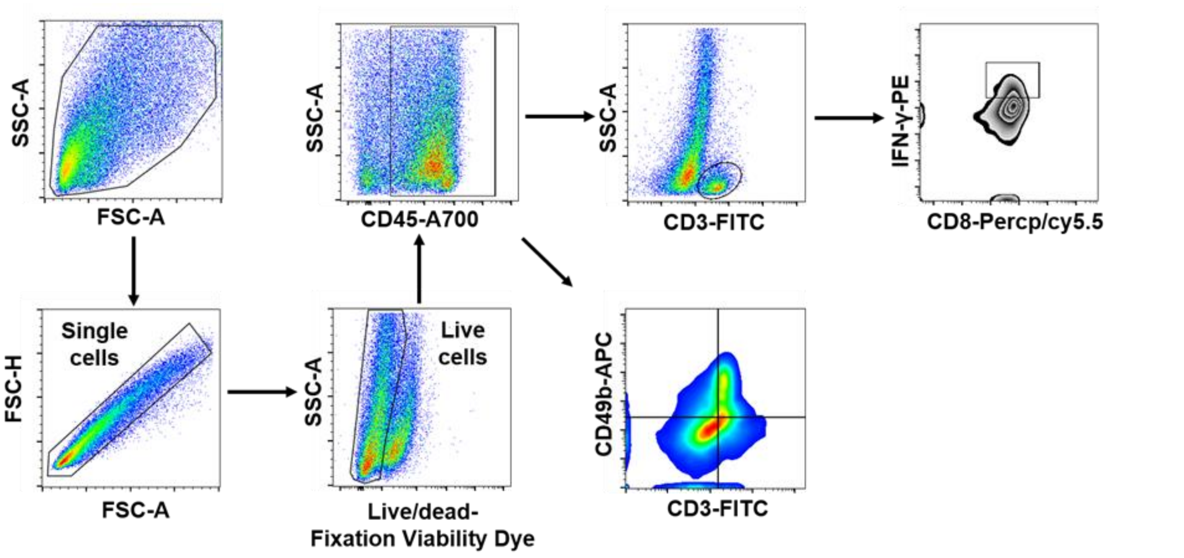

Figure S27. Gating strategy for analysis of IFN-γ^+ effector $CD4^+$ T cells and NK cells ($CD45^+CD3^-CD49b^+$) in tumor.

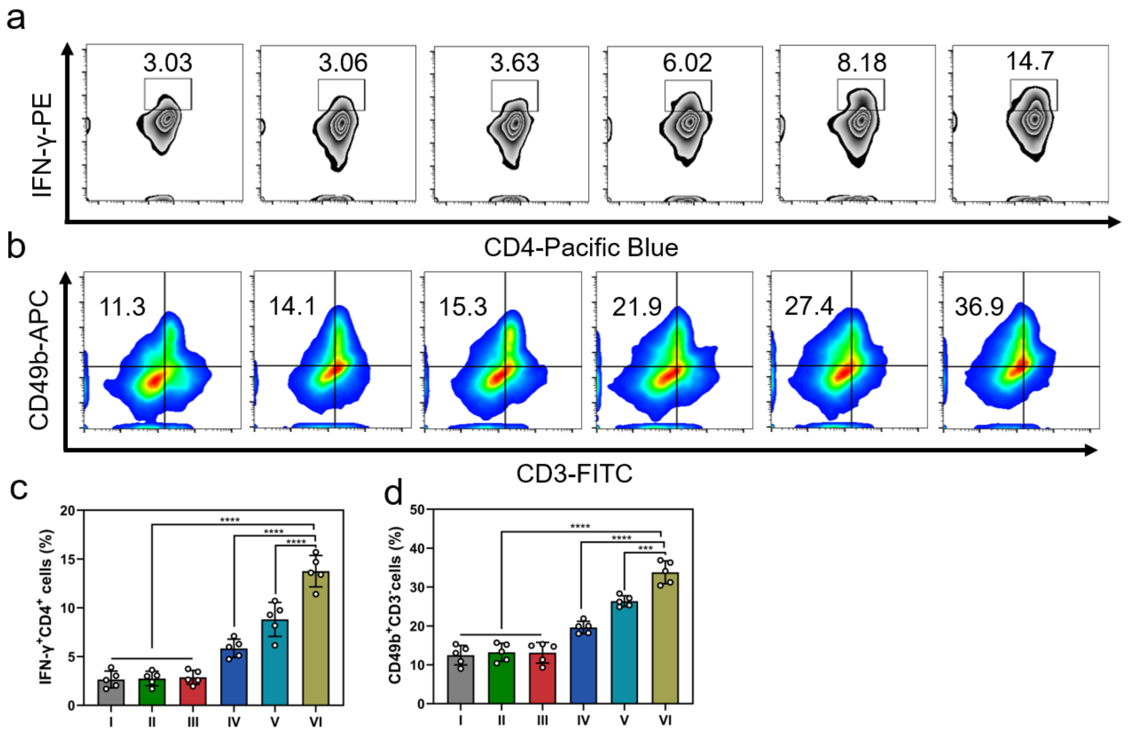

Figure S28. (a, b) Representative flow cytometry plots of IFN-γ^+ effector $CD4^+$ T cells and NK cells ($CD45^+CD3^-CD49b^+$) in tumor. (c, d) Quantitative analysis of the percentages of IFN-γ^+ effector $CD4^+$ T cells and NK cells ($CD45^+CD3^-CD49b^+$). Data are shown as the mean ± SEM (n=5). (*P < 0.05, **P < 0.01, ***P < 0.001, ****P < 0.0001).

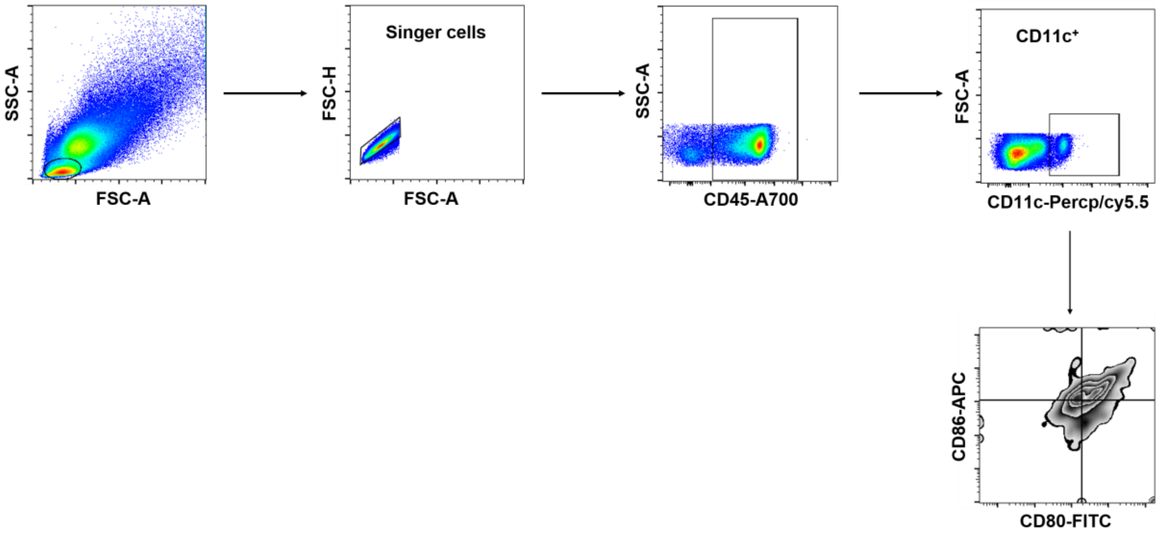

Figure S29. Gating strategy for analysis of dendritic cells (DCs) in spleen and tumor-draining lymph node (TDLN). (n = 5).

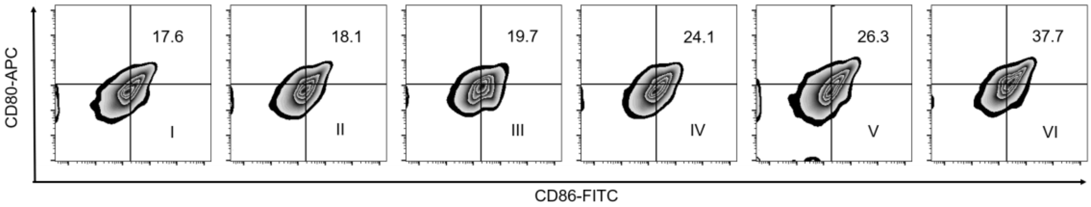

Figure S30. Flow cytometric analyses of the population of DCs in TDLN (n = 5).

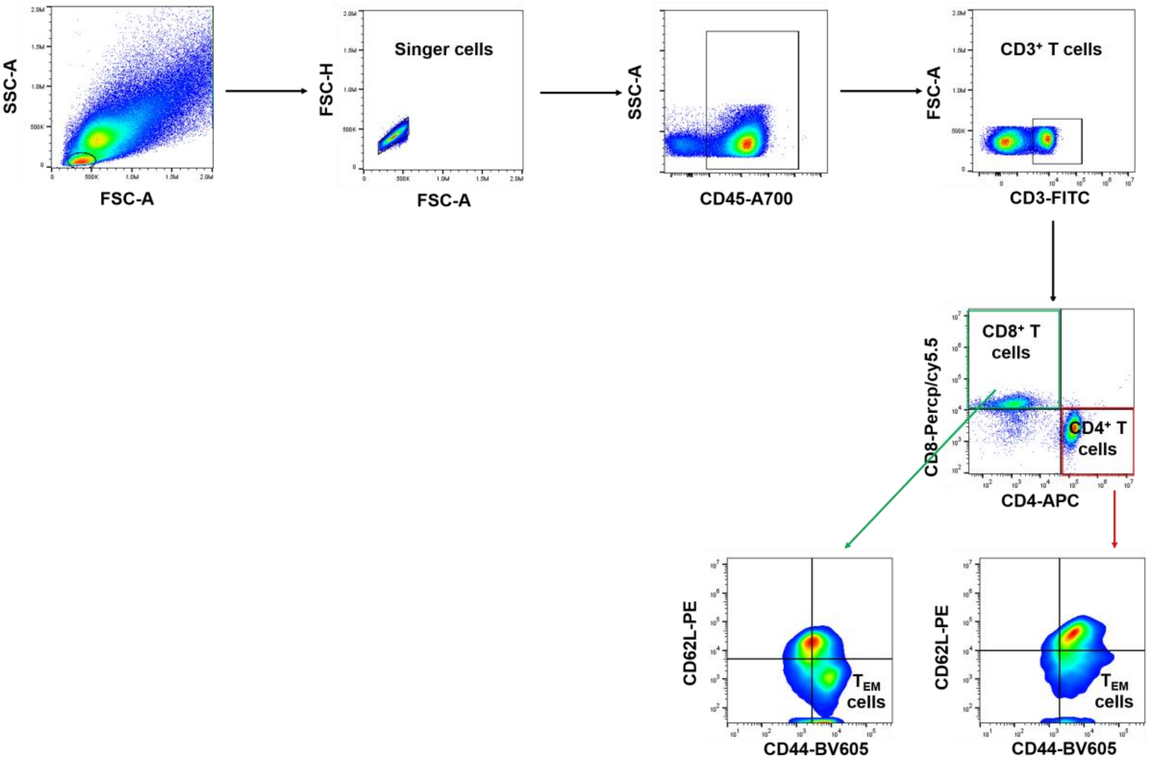

Figure S31. Gating strategy for analysis of effector memory T cells in spleen and TDLN. The effector memory T cells (TEM) were gated on $CD3^{+}CD4^{+}CD62L^{-}CD44^{+}$ cells. (n = 5).

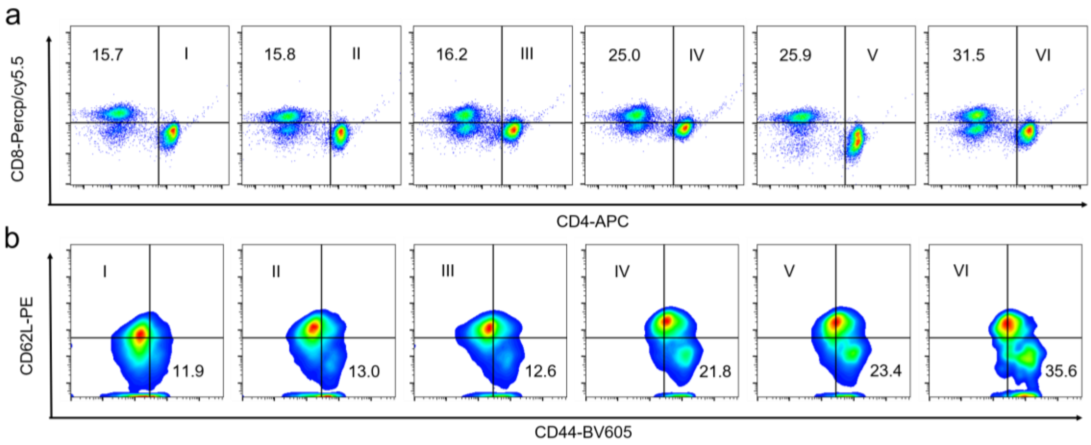

Figure S32. Flow cytometric analyses of the population of (a) $CD4^+$ and $CD8^+$ T cells, (b) effector-memory (EM) cells in $CD4^+$ T cells in splenocytes. (n = 5).

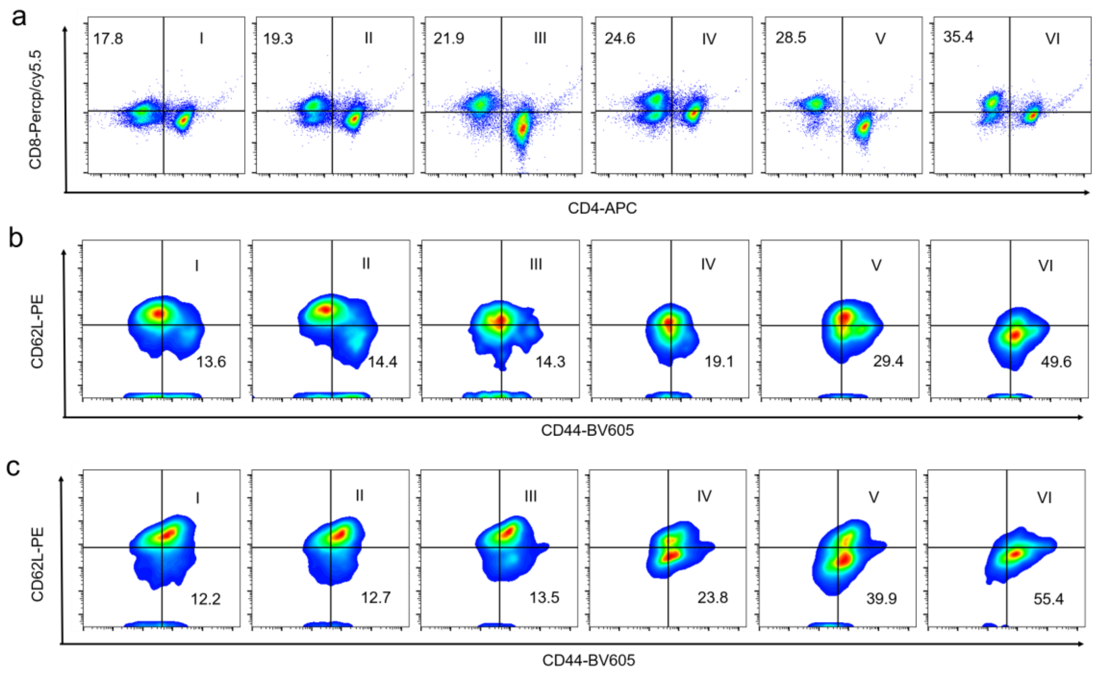

Figure S33. Flow cytometric analyses of the population of (a) $CD4^+$ and $CD8^+$ T cells, (b) EM cells in $CD4^+$ T cells, (c) EM cells in $CD8^+$ T cells in TDLN (n = 5).

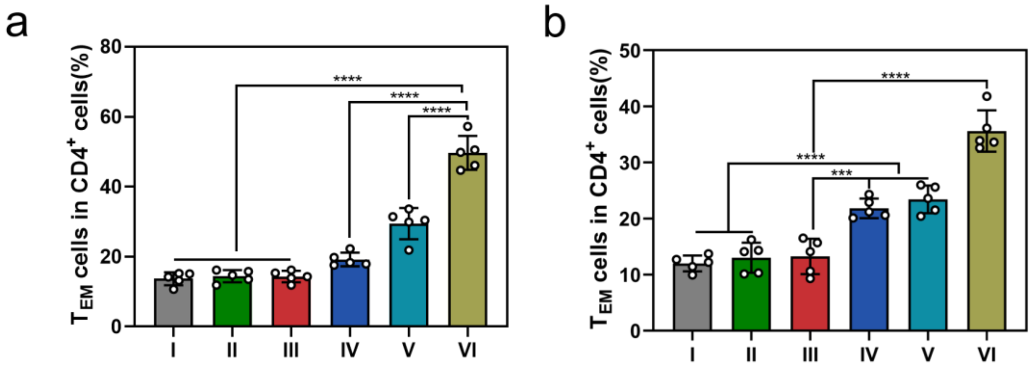

Figure S34. Percentages of EM cells in $CD4^+$ T cells in spleen and TDLN were analyzed by Flow cytometry on day 22 after tumor incubation (n = 5). Data are shown as the mean ± SEM. (*P < 0.05, **P < 0.01, ***P < 0.001, ****P < 0.0001).
